# Prognostic Indicators for Precision Treatment of Non-Small Cell Lung Carcinoma

**DOI:** 10.3390/cells13211785

**Published:** 2024-10-28

**Authors:** Damayanti Das Ghosh, Hannah McDonald, Rajeswari Dutta, Keerthana Krishnan, Jaya Thilakan, Manash K. Paul, Neha Arya, Mahadev Rao, Vivek M. Rangnekar

**Affiliations:** 1 Basic and Translational Research Division, Saroj Gupta Cancer Centre and Research Institute, Mahatma Gandhi Road, Kolkata 700063, West Bengal, India; damayantidas@gmail.com (D.D.G.); rajeswaridutta18@gmail.com (R.D.); 2 School of Health Sciences and Translational Research, Sister Nivedita University, Newtown, Kolkata 700156, West Bengal, India; 3 Department of Surgery, University of Kentucky, Lexington, KY 40536, USA; hannah.mcdonald@uky.edu; 4 Department of Pharmacy Practice, Manipal College of Pharmaceutical Sciences, Manipal Academy of Higher Education, Manipal 576104, Karnataka, India; keerthana.krishnan1@learner.manipal.edu; 5 Department of Biochemistry, All India Institute of Medical Sciences Bhopal, Bhopal 462020, Madhya Pradesh, India; jthilakan5@gmail.com; 6 Department of Genetics, UTD, Barkatullah University Bhopal, Bhopal 462026, Madhya Pradesh, India; 7 Department of Radiation Biology and Toxicology, Manipal School of Life Sciences, Manipal Academy of Higher Education, Manipal 576104, Karnataka, India; manash.paul@manipal.edu; 8 Department of Translational Medicine, All India Institute of Medical Sciences Bhopal, Bhopal 462020, Madhya Pradesh, India; neha.tmc@aiimsbhopal.edu.in; 9 Markey Cancer Center and Department of Radiation Medicine, University of Kentucky, Lexington, KY 40536, USA

**Keywords:** prognostic marker, precision medicine, lung cancer, therapy resistance

## Abstract

Non-small cell lung cancer (NSCLC) has established predictive biomarkers that enable decisions on treatment regimens for many patients. However, resistance to therapy is widespread. It is therefore essential to have a panel of molecular biomarkers that may help overcome therapy resistance and prevent adverse effects of treatment. We performed in silico analysis of NSCLC prognostic indicators, separately for adenocarcinomas and squamous carcinomas, by using The Cancer Genome Atlas (TCGA) and non-TCGA data sources in cBioPortal as well as UALCAN. This review describes lung cancer biology, elaborating on the key genetic alterations and specific genes responsible for resistance to conventional treatments. Importantly, we examined the mechanisms associated with resistance to immune checkpoint inhibitors. Our analysis indicated that a robust prognostic biomarker was lacking for NSCLC, especially for squamous cell carcinomas. In this work, our screening uncovered previously unidentified prognostic gene expression indicators, namely, *MYO1E*, *FAM83* homologs, and *DKK1* for adenocarcinoma, and *FGA* and *TRIB1* for squamous cell carcinoma. It was further observed that overexpression of these genes was associated with poor prognosis. Additionally, *FAM83* homolog and *TRIB1* unexpectedly harbored copy number amplifications. In conclusion, this study elucidated novel prognostic indicators for NSCLC that may serve as targets to overcome therapy resistance toward improved patient outcomes.

## 1. Introduction

**Figure 1:**
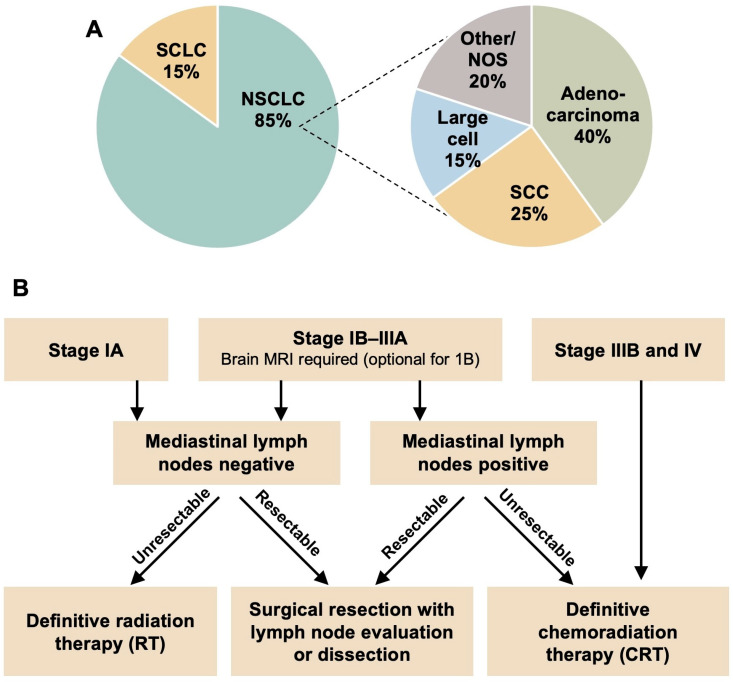
Lung cancer types and treatment regimens. (**A**) Lung cancer types with corresponding frequency of incidence (%) are divided into small cell lung cancer (SCLC) and non-small cell lung cancer (NSCLC). The subtypes of NSCLC are listed in the right panel. (**B**) Treatment of NSCLC by disease stage according to NCCN guidelines. NOS: Not Otherwise Specified; MRI: Magnetic Resonance Imaging.

The Global Cancer Observatory has reported 19.9 million cases and 9.7 million cases of incidence and death worldwide. In particular, lung cancer is the most frequently detected cancer with the highest incidence of 2.5 million cases and mortality of 1.8 million cases worldwide [1]. At the histological level, lung cancer can be classified into two major subtypes: non-small cell lung cancer (NSCLC), which corresponds to 85% of all lung cancers, and small cell lung cancer (SCLC), which corresponds to 25% of lung cancers. In addition, NSCLC can be further subdivided into adenocarcinoma (40%), squamous cell carcinoma (25%), large cell carcinoma (15%), and other/not otherwise specified (NOS, 20%) (Figure 1A). About 50–60% of patients are diagnosed with an advanced stage of lung cancer, i.e., stage IV as per the TNM staging, 20–25% are diagnosed as locally advanced lung cancer, i.e., stage III as per the TNM staging, and 20–25% are diagnosed at an early stage, i.e., stage I/II as per the TNM classification.

Risk factors for the development of NSCLC are both environmental and genetic. While the environmental causes include tobacco exposure, radon, asbestos, and chronic inflammatory conditions of the lungs, the genetic causes may be inherited germline mutations or (most commonly) acquired somatic mutations [2]. Many mutations (mut.), amplifications (amp.), and rearrangements (rearr) have been implicated in the development of aggressive NSCLC, including *KRAS* (mut), *EGFR* (mut), *FGFR1* (amp), *ALK* (rearr), *HER2* (mut), *MET* (amp), and many other rare alterations in *BRAF*, *PIK3CA*, *AKT1*, *MEK1*, *NRAS*, *RET*, *RB1*, and *NTRK* (Table 1) [2]. Genomic testing is critical for the diagnosis and treatment of NSCLC, as many of these alterations confer resistance and/or susceptibility to first- or second-line therapies. The standard of care therapy in NSCLC depends on the stage of the disease (Figure 1B). Surgery is the gold standard for resectable, stage I disease, and must include systematic/mediastinal lymph node dissection. For unresectable tumors, definitive radiotherapy is usually recommended. In cases of larger tumor size (>1 cm) or lymph node involvement, systemic chemotherapy is always considered (https://www.nccn.org/professionals/physician_gls/pdf/nscl.pdf; accessed on 29 August 2024). Systemic therapy consists of a combination of chemotherapy and immune checkpoint inhibitors (ICIs). The choice of chemotherapy depends on histology and may include platinum doublet therapy such as carboplatin vs. cisplatin/paclitaxel, among others. Patients who are without contraindications to ICI therapy will also receive anti-PD-1 antibody, nivolumab. As resistance develops, second-line therapies used are tyrosine kinase inhibitor (TKI) osimertinib, and ICIs atezolizumab and pembrolizumab, in the absence of contraindications (https://www.nccn.org/professionals/physician_gls/pdf/nscl.pdf) [3]. Among patients with NSCLC, a significant proportion (~64%) who initially respond positively to ICI exhibit acquired resistance to second-line ICI therapy [4]. Despite several studies aiming to uncover biomarkers that might indicate primary unresponsiveness, there remains a significant gap in our understanding. Therefore, there is a need to develop better predictive markers that can be used for appropriate decision making, and it demands a better understanding of resistance to standard treatments.

Resistance to therapy in NSCLC can develop via multiple different mechanisms and is attributed to molecular alterations, primarily mutations in key oncogenic drivers. Therefore, progression on first- or second-line therapy should prompt specialized genomic testing to evaluate for alterations known to affect response to therapy. These tests may also identify alterations that are known to confer higher vulnerability to the tumor-targeted therapies. This review describes the limitations associated with current treatment protocols in NSCLC and in silico identification of potential biomarkers for improved clinical decision making.

## 2. Targeted Therapy Resistance in NSCLC

Targeted therapy is an important treatment regimen in NSCLC. While targeted therapy is the mainstay, and increased response is observed in patients with NSCLC, cases of tumor recurrence have been reported. One of the major factors responsible for tumor recurrence or increased mortality is drug resistance. This section will discuss the resistance associated with the current targeted therapy for NSCLC.

## 3. TKI Resistance

TKIs, such as erlotinib, gefitinib, afatinib, and osimertinib, are approved as targeted therapy for NSCLC with *EGFR* mutations. EGFR is a transmembrane tyrosine kinase receptor that plays a critical role in cell survival, growth, and division following the binding of EGFR to its ligands, namely, epidermal growth factor and transforming growth factor-alpha and subsequent upregulation of downstream signaling pathways such as protein kinase B and mitogen-activated protein kinases. *EGFR* activation-based mutation or amplification is correlated with initiation, progression, and poor prognosis of NSCLC [5]. *EGFR* mutation contributes to approximately 50% of mutations in Asian subjects with adenocarcinoma [6]. Therefore, the gold standard treatment of patients with NSCLC and harboring *EGFR* mutation is targeted therapy. EGFR tyrosine kinase inhibitors (TKIs) have demonstrated good initial response in patients with *EGFR* mutations; however, the majority exhibit drug resistance leading to disease progression. More specifically, patients who were administered targeted therapy based on first- and second-generation TKIs, such as erlotinib and gefitinib, reported the development of acquired drug resistance within 9–14 months [7]. About 50–70% of patients develop T790M mutation, which is characterized by substitution of threonine to methionine at amino acid position 790 in exon 20 [8,9,10]. At the molecular level, T790M mutation affects TKI binding to EGFR’s ATP-binding site, thereby inhibiting downstream signaling cascade. T790M mutation was solved with the advent of third-generation TKIs, such as osimertinib [11,12,13,14]. AURA serial studies reported the effectiveness and manageable safety of osimertinib when administered to T790M mutation-positive advanced NSCLC patients [15]. However, like first- and second-generation TKIs, patients administered with osimertinib have demonstrated acquired resistance such as G796D and C797S [14,16].

In addition, rearrangements in *ALK* and *ROS1* lead to their constitutive oncogenic expressions, which can be inhibited by the drug crizotinib [17,18]. ALK, a member of the insulin receptor superfamily, is a transmembrane receptor tyrosine kinase and its expression is seen during embryogenesis in the nervous system and declines postnatally. Rearrangement in *ALK* is seen in approximately 6.7% of patients with NSCLC [19]. Additionally, *ALK* fusion has been clinically observed in younger patients with adenocarcinoma with no or infrequent history of smoking; the most common type of fusion reported is *EML4-ALK* [20]. ALK-TKIs have demonstrated success in advanced cases of *ALK*-positive NSCLC; however, the mutation in the ALK kinase domain leads to secondary resistance in targeted treatment, with G1202R being the most common mutation.

On the other hand, mutations in ROS1, a member of the transmembrane tyrosine kinase family, typically contribute to approximately 1–2% mutations in NSCLC. It is also observed in patients who do not smoke or infrequently smoke. The most common fusion partners of the *ROS1* gene include *CD74*, *EZR*, *SDC4*, and *SLC34A2* [21,22]. It has further been reported that typically *ROS1* rearrangement does not coexist with a mutation in other genes, including *EGFR*, *ALK*, and *KRAS*; however, recent rare studies have reported *ROS1* mutation in conjunction with *EGFR* and *ALK* mutations [23,24]. The first FDA-approved targeted treatment for *ROS1*-positive NSCLC was crizotinib [25]; G2032R is the most common drug resistance mutation and has clinically responded to ROS1/TRK/ALK inhibitors such as repotrectinib and taletrectinib [25].

Other studies have demonstrated crizotinib resistance due to L1196M mutation in *ALK* or EGFR/KRAS pathway activation, which is otherwise mutually exclusive with *ALK* rearrangement [26]. Towards this, multi-kinase inhibitors ceritinib, alectinib, brigatinib, and lorlatinib are known to treat crizotinib-resistance in *ALK* or *ROS1* rearranged NSCLCs [27,28]. Dabrafenib is a *BRAF* inhibitor used in combination with trametinib (*MEK* inhibitor) in metastatic NSCLC bearing V600E [29]. The FDA has recently approved capmatinib and tepotinib resulting from the GEOMETRY mono-1 and VISION trials, respectively, for metastatic NSCLC [30,31]. The FDA has also approved selpercatinib and pralsetinib based on results of LIBRETTO-001 and ARROW trials, respectively, for *RET*-altered NSCLC [32]. Larotrektinib (pan-NTRK inhibitor) has been FDA-approved for the treatment of *NTRK*-altered NSCLC because of several trials like LOXO-TRK-14001, SCOUT, and NAVIGATE [27]. The multi-kinase inhibitor cabozantinib is in a phase II clinical trial for *ROS1* and *NTRK*-altered NSCLC, and seribantumab, a monoclonal antibody, is being tested for *NRG1*-altered advanced NSCLC [27,33]. Phase III trials are ongoing to test combination therapies against *KRAS*-mutated NSCLC [34]. In addition, several drugs are being tested against *HER2* alterations, including afatinib in a clinical trial called NICHE, and trastuzumab/deruxtecan combination in another trial called DESTINY-Lung01 [35,36].

Another etiology for TKI resistance to therapy is the transformation of NSCLC (about10%) to small cell lung cancer (SCLC), which can be seen concurrently with *EGFR* alterations [37,38,39] (https://www.nccn.org/professionals/physician_gls/pdf/nscl.pdf; accessed on 29 August 2024). Small cell transformation (SCT) may be explained by either of the two hypotheses. In this regard, the pseudo-SCT hypothesis states that some TKI-resistant NSCLCs may harbor a small component of SCLCs which may become predominant after TKI treatment. The more accepted second hypothesis portrays the descent of lung adenocarcinomas (LUAD) and SCLCs from identical alveolar type II cells, and that NSCLC can trans-differentiate into SCLC [37]. To this, earlier studies reported the role of TP53- and RB-deficiency in the lineage plasticity of cells [40]. Unlike original SCLCs, transformed SCLCs retain *EGFR* mutations [38].

In conclusion, while TKIs have demonstrated success in the management of NSCLC, there is a need to develop better therapies to manage secondary drug resistance mutations.

## 4. Immune Checkpoint Inhibitor Resistance

One of the major advancements in the field of cancer immunotherapy is the introduction of immune checkpoint inhibitors (ICIs). ICIs are typically represented by monoclonal antibodies blocking cytotoxic T-lymphocyte-associated protein 4 (CTLA-4), programmed cell death 1 (PD-1), and programmed cell death-ligand (PD-L1). ICIs help the immune system identify cancer cells as foreign bodies and counteract the immune system’s tumor-driven inhibition that fosters tumor growth. The ICI-mediated survival benefit has established it as first-line therapy in patients with advanced or locally advanced NSCLC and extensive SCLC. Approximately 15–20% display partial or complete response [41]. The efficacy of immune checkpoint inhibitors (ICIs) is modulated by multiple underlying gene mutations. As an example, increased efficacy has been reported with *BRAF* and *MET* alterations, whereas resistance has been observed with *ALK* rearrangements and *EGFR* (exon 19 deletion vs. exon 21p.L858R) mutations. For this reason, tumors with the latter two alterations bear a relative contraindication for ICI therapy [42,43] (https://www.nccn.org/professionals/physician_gls/pdf/nscl.pdf; accessed on 29 August 2024).

Currently approved ICIs include ipilimumab against CTLA-4, nivolumab and pembrolizumab against PD-1, and atezolizumab, durvalumab, and avelumab against PD-L1 [44,45]. CTLA-4 and PD-1 are expressed on T cells, while PD-L1 is expressed on tumor cells. It has also been shown that the ICIs work well in the presence of tumor-infiltrating immune cells [46]. The anti-tumor immune response depends on innate immunity-driven pro-inflammatory chemokines and proper antigen presentation by tumor cells for inducing specific immunity. The cytotoxic T-cell (CTL) response is driven by antigen-presenting cells (APCs). APCs present tumor antigens to the CD8+ Tc cells via their human leukocyte antigen (HLA) to the T-cell receptors (TCR) of CD8+ Tc cells along with co-stimulatory signals. Sub-optimal performance in any of these pathways may inhibit anti-tumor immunity and confer ICI resistance [45].

The lack of tumor-infiltrating lymphocytes (TILs) in the tumor immune microenvironment (TIME) is strongly associated with ICI resistance [47]. The tumor immune microenvironment (TIME) can be classified as T1 (TIL−, PD-L1−), T2 (TIL+, PD-L1+), T3 (TIL−, PD-L1+), and T4 (TIL+, PD-L1−), of which, T2 is most responsive to ICIs [29]. Reportedly, only 17% of NSCLCs have been shown to possess the T2 phenotype [45]. The absence of T2, or IFN-γ deficiency in T3, or lack of PD-L1 in T1, ectopic expression of PD-L1 on tumor-associated macrophages/platelets, or presence of driver mutations in *EGFR*/*ALK*/*ROS1*/*MET*/*RET*, or presence of secreted PD-L1, forms sets of different probable events leading to ICI-resistance [48,49,50,51,52,53,54].

Reduced HLA class I expression, impaired β2-microglobulin mediated HLA class I stability, or presence/absence of specific HLA class I alleles are associated with ICI resistance [55,56,57]. ICI resistance can also result from the prevention of immune over-activation by other co-inhibitory molecules like LAG-3, TIM-3, and TIGIT expressed on various immune cells, as well as non-classical HLA-G and HLA-E expressed on tumor cells [58,59,60,61]. IFN acts through the JAK/STAT pathway, and loss-of-function mutations in *JAK1/2* or *STAT1*-related epigenomic changes can induce ICI resistance and T-cell inhibition [62,63]. Taken together, the underlying mechanisms associated with ICI resistance demand a better understanding for guiding drug development and improved precision therapy approaches.

## 5. Biomarkers for Lung Cancer

Cancer-related biomarkers are characteristic proteins or nucleic acids derived from blood or affected tissues of cancer patients that help to detect the disease and indicate response to treatment or survival patterns in patient cohorts [27]. These markers are of three main types: (a) diagnostic markers that help disease detection, (b) predictive markers that enable treatment effectiveness, and, (c) prognostic markers that allow prediction of clinical outcomes unrelated to treatment [27]. Even though advancements in lung cancer therapeutics continue to rise, the overall prognosis remains poor. While various markers including age, performance status, disease stage, and grade risk-stratify lung cancer patients with improved clinical decision making, there is a need to develop sensitive and specific biomarkers that can provide better prognosis status as well as clinical decision making.

Our analysis has revealed that treatment options for NSCLC have become sparse due to therapeutic resistance. Moreover, there is a lack of prognostic biomarkers for NSCLC. Hence, generating a more comprehensive understanding of a larger pool of predictive/prognostic biomarkers that can be readily available for clinical trials or treatment is important. The upcoming sections will discuss the various predictive and prognostic markers reported for NSCLC. It was also observed that predictive/prognostic biomarkers are primarily available for LUAD, but not for lung squamous cell carcinomas (LUSC). Therefore, this review identifies novel prognostic biomarkers for lung adenocarcinomas, as well as lung squamous carcinomas, by harnessing the University of Alabama at Birmingham CANcer (UALCAN) data portal as well as The Cancer Genome Atlas (TCGA)-based and non-TCGA data sources from cBio Portal [64,65] (https://www.cbioportal.org/; accessed on 29 August 2024).

## 6. Predictive Markers for Lung Cancer

*Non-genetic markers:* Tumor histology is the most used non-genetic predictive marker for NSCLC. In this context, pemetrexed treatment is beneficial for non-squamous but not squamous cell histology of NSCLC [66].

*Genetic markers:* The guidelines by The College of American Pathologists (CAP), the International Association for the Study of Lung Cancer (IASLC), and the Association for Molecular Pathology (AMP) recommend routine multigene testing of all advanced NSCLC with an adenocarcinoma component. Toward this, the genetic alterations implicated in NSCLC involve exon 19 deletions, exon 21 substitution (L858R), exon 20 insertion, L861Q, G719X, and S768I of *EGFR*; rearrangement, insertion, and point mutations of *ALK*; rearrangements of *ROS1*, *RET*, *NTRK*, and *NRG1*; exon 14-skipping mutation and amplification of *MET*; overexpression of PD-1 or PD-L1; G12C/V/D/A point mutations in *KRAS*; and homo/hetero-dimerization, amplification of HER2 or mutations in *HER2/ERBB* [27,67,68]. These tests are also recommended for younger, non-smoker patients with squamous cell carcinomas. The significance of the mutations in exons 18, 19, and 21, with the concurrent absence of T790M, has demonstrated a direct correlation with better response with TKI treatment. Further, the presence of T790M and C797S shows resistance against first/second and third generations of TKI, respectively. Routine testing for T790M mutation in *EGFR* is performed in all patients with activating *EGFR* mutations with evidence of EGFR-TKI resistance [69]. In addition, the current Pan-Asian guidelines recommend PD-L1immunohistochemistry in all patients with advanced non-squamous type of NSCLC along with the tests [70]. The significance of PD-L1 overexpression lies in being druggable with immunotherapy and associated with longer survival. However, these markers can predict only therapeutic responses. Despite the available predictive markers and associated therapeutic regimens, therapeutic resistance is common in NSCLC.

### 6.1. Genes and Pathways Involved

The biomarkers established in the prediction of response to targeted therapy for non-squamous NSCLC are the receptor tyrosine kinases, *EGFR*, *ALK*, and *ROS1*, along with *RET*, *MET*, *HER2*, *KRAS*, *BRAF*, *NTRK*, and *NRG1*. The predictive biomarkers for response to immunotherapy include PD-1 and PD-L1 overexpression. EGFR, also called HER1, and KRAS, a GTPase, are involved in cellular signal transduction by Raf/MEK/ERK, PI3K/AKT/mTOR, and RalGDS/RalAIB pathways, and interestingly, their mutations are mutually exclusive [71]. Specific activating/sensitizing mutations in the tyrosine kinase domain of EGFR drive constitutive expression of the gene leading to tumorigenic, uncontrolled cell proliferation. ALK, also called CD246, signals PI3K/AKT, RAS/MAPK, and JAK/STAT pathways, while ROS1 drives cell growth and differentiation [17,72]. BRAF is a cytosolic serine/threonine kinase downstream of KRAS that drives the MAPK pathway [73]. MET, RET, NTRK, NRG1, and HER2/ERBB are receptor tyrosine kinases driving similar pathways as EGFR or KRAS. PD-1, also called B7-H1, is expressed by different immune cells like B and T lymphocytes, macrophages, and antigen-presenting cells. PD-1 acts as the receptor for PD-L1 expressed on activated cytotoxic T cell surfaces and on lung cancer cells. The binding causes suppression of the immune system and immune evasion of tumor cells [74,75].

### 6.2. Histology and Population-Specific Statistics

The most frequent mutations in NSCLC include exon 19 deletion (45–50% of all *EGFR* mutated cases) and exon 21 substitution (L858R) (35–45% of all *EGFR* mutated cases) that are commonly found in non-smoking women from East Asia with adenocarcinoma [76]. Exon 19 deletion and exon 21 substitution are less frequent in squamous cell carcinoma, i.e., among 3.3% and 4.6% of Western and Asian populations, respectively [27]. Other frequent mutations comprise *EML4* inversion rearrangement with *ALK* generating *EML4-ALK* fusion oncogene that is found in 2–7% of NSCLC cases who are mainly non-smoker adenocarcinoma patients of a median age of 55 years [28,77]. The most frequent *BRAF* mutation is the V600E constitutively activating point mutation found in 2–4% of adenocarcinomas, which is mutually exclusive with *KRAS* mutations [73,78]. Non-V600E mutations are reported in male smokers and can co-exist with *KRAS* mutations [78,79]. *ROS1* rearrangement is found in 1–3% of adenocarcinomas, with *CD74-ROS1* fusion being the most common [72,80]. About 1–2% of NSCLCs, primarily adenocarcinomas, harbor *RET* fusions, of which the most common is *RET-KIF5B* fusion [81,82,83]. In addition, the most common *NTRK* fusions are *MPRIP-NTRK1* and *CD74-NTRK1*, found in 3% of adenocarcinomas [84]. *NRG1* fusions are found in around 0.3% of NSCLCs, of which *CD74-NRG1* fusion is the most found mutation [85]. Additionally, activating *KRAS* mutations are also found in adenocarcinomas more predominantly in the West (30%) than in Asia (10%) [86]. HER2 overexpression occurs in 2–38% of adenocarcinomas and in 1–16% of squamous carcinomas, while *HER2* mutations appear in 2% of cases, with exon 20 in-frame insertion being the most frequent type predominantly found among non-smoker females [87,88].

## 7. Prognostic Markers for Lung Cancer

Non-genetic prognostic markers comprise TNM stage, patient age, gender, and performance status. TNM is based on tumor size (T), nodal involvement (N), and distant metastasis (M) [89]. The performance score is based on the level of functionality and self-care, and is measured by tools such as ECOG (Eastern Cooperative Oncology Group) and KPS (Karnofsky performance status) [90]. On the other hand, *genetic* prognostic markers for NSCLC are non-conventional molecular alterations that have shown promising value in preclinical experiments [91]. An earlier report categorized ‘good prognosis’ and ‘bad prognosis’ groups separately for adenocarcinoma and squamous carcinoma based on expression patterns of sets of six proteins (c-SRC, CCNE1, TTF1, P65, CHK1, and JNK1/MPK1) and five proteins (EGFR, SOX2, CDH1, AKT1, and MAPK14), respectively [92]. Another report showed that lower protein expression of Girdin/CCDC88A and STAT3 correlated with longer survival than higher expression [93]. In addition, higher expression of the proliferation marker, MKI67, was found to be correlated with higher TNM stage and recurrence [94]. Furthermore, higher expression of PINK1, involved in mitophagy, was associated with chemoresistance and found to be a perilous prognostic factor in adenocarcinoma, but not for squamous cell carcinoma [95,96]. Another study based on specific immunohistochemical markers found that CK20-positive, CDX2-positive, and CEA-negative adenocarcinoma cases correlated with better survival [96]. The expression pattern of 20 out of 1500 candidate genes, namely, *CERS4*, *FUT4*, *C3ORF18*, *CYP17A1*, *ASPM*, *HJURP*, *LOC645166*, *DENND1C*, *SLC25A42*, *CCNA2*, *LDHA*, *IGFBP1*, *SLC2A1*, *DAAM2*, *RGS20*, *MFI2*, *LDLRAD3*, *KLHDC8B*, *CREG2*, and *SPATA6*, were also associated with prognostication in NSCLC [97]. Other studies have similarly shown high expression of *VEGF*, *TUBB3*, and *TGFB1*, and low expression of *ERCC1* to be separately associated with poor prognosis in NSCLC [27]. Although mutated *TP53*, which is more frequent in squamous cell carcinomas (77%) than adenocarcinomas (47%), has shown worse prognosis and therapy resistance in NSCLC [98,99,100], some studies failed to validate its prognostic power [101].

In addition, many non-coding RNAs have been reported to show prognostic implications in NSCLC. Toward this, low expression of miR-590, miR-340, and miRNA-125a-5p, deregulated miR-155 expression in adenocarcinomas, and high expression of miR-494-3p were found to be hazardous [102,103,104,105,106]. Further, there are reports that inhibition of circ_001569 (circular RNA) and low expression of LINC00152 (Non-coding RNA-Cytor or Cytoskeleton Regulator RNA) improved survival [107,108]. A panel of five long non-coding RNAs (lncRNA), namely, FLJ30679, LINC00511, LINC01127, MIF-AS1, and RP11-278J6.4, were reported as poor prognostic factors for NSCLC [109]. Additionally, the tumor mutation burden, neutrophil to lymphocyte ratio, and platelet to lymphocyte ratio are potential prognostic markers [27]. In the upcoming section, the ongoing research on prognostic biomarkers has been showcased based on individual studies that are yet to be clinically validated.

## 8. InSilicoSearch for Prognostic Biomarkers Using UALCAN

**Figure 2:**
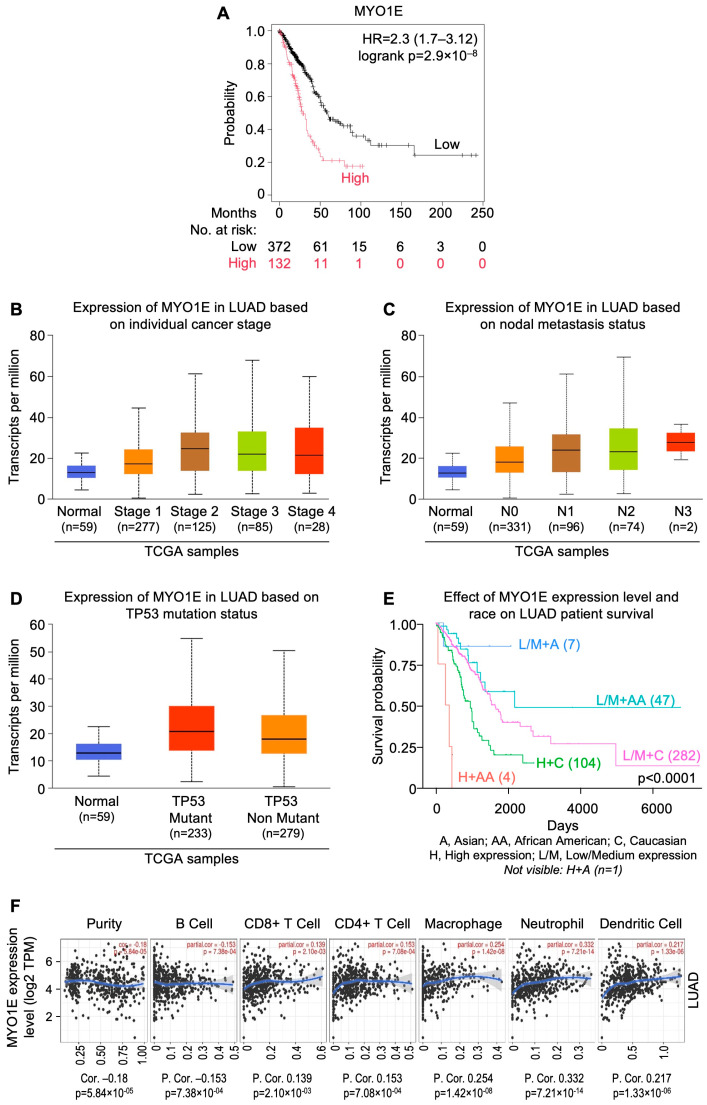
*MYO1E* gene expression as a candidate prognostic indicator for lung adenocarcinomas from UALCAN-based analysis of TCGA data. (**A**) Relation between gene expression and overall survival as depicted by KM Plotter. (**B**) Relation between gene expression and disease stage. (**C**) Relation between gene expression and metastatic status. (**D**) Relation between gene expression and *TP53* mutation status. (**E**) Relation between gene expression, ethnicity, and overall survival. (**F**) Correlation of gene expression with tumor infiltrating lymphocytes using TIMER 2.0. [LUAD: lung adenocarcinomas; NSCLC: non-small cell lung carcinoma; TCGA: The Cancer Genome Atlas; UALCAN: University of Alabama at Birmingham CANcer data analysis Portal].

**Figure 3:**
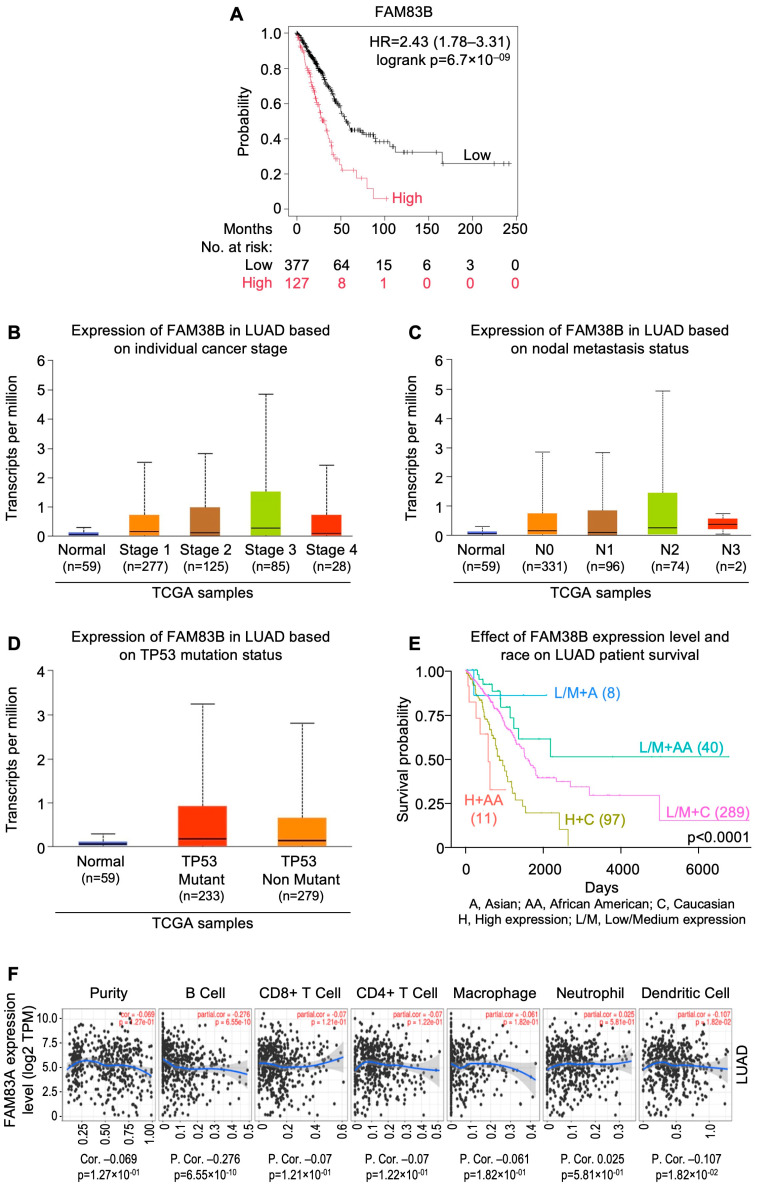
*FAM83* homologs: gene expression as a candidate prognostic indicator for lung adenocarcinomas from UALCAN-based analysis of TCGA data. (**A**) Relation between gene expression and overall survival as depicted by KM Plotter. (**B**) Relation between gene expression and disease stage. (**C**) Relation between gene expression and metastatic status. (**D**) Relation between gene expression and *TP53* mutation status. (**E**) Relation between gene expression, ethnicity, and overall survival. (**F**) Correlation of gene expression with tumor infiltrating lymphocytes using TIMER 2.0. [LUAD: lung adenocarcinomas; NSCLC: non-small cell lung carcinoma; TCGA: The Cancer Genome Atlas; UALCAN: University of Alabama at Birmingham CANcer data analysis Portal].

**Figure 4:**
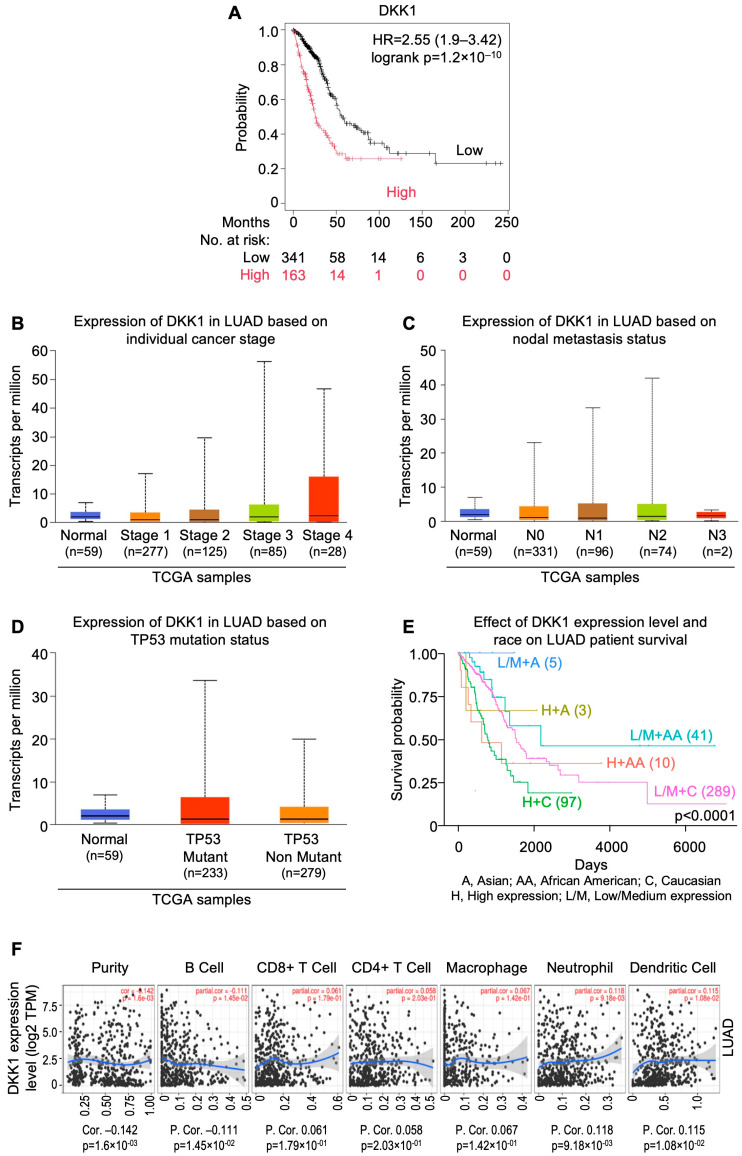
*DKK1* gene expression as a candidate prognostic indicator for lung adenocarcinomas from UALCAN-based analysis of TCGA data. (**A**) Relation between gene expression and overall survival as depicted by KM Plotter. (**B**) Relation between gene expression and disease stage. (**C**) Relation between gene expression and metastatic status. (**D**) Relation between gene expression and *TP53* mutation status. (**E**) Relation between gene expression, ethnicity, and overall survival. (**F**) Correlation of gene expression with tumor infiltrating lymphocytes using TIMER 2.0. [LUAD: lung adenocarcinomas; NSCLC: non-small cell lung carcinoma; TCGA: The Cancer Genome Atlas; UALCAN: University of Alabama at Birmingham CANcer data analysis Portal].

**Figure 5:**
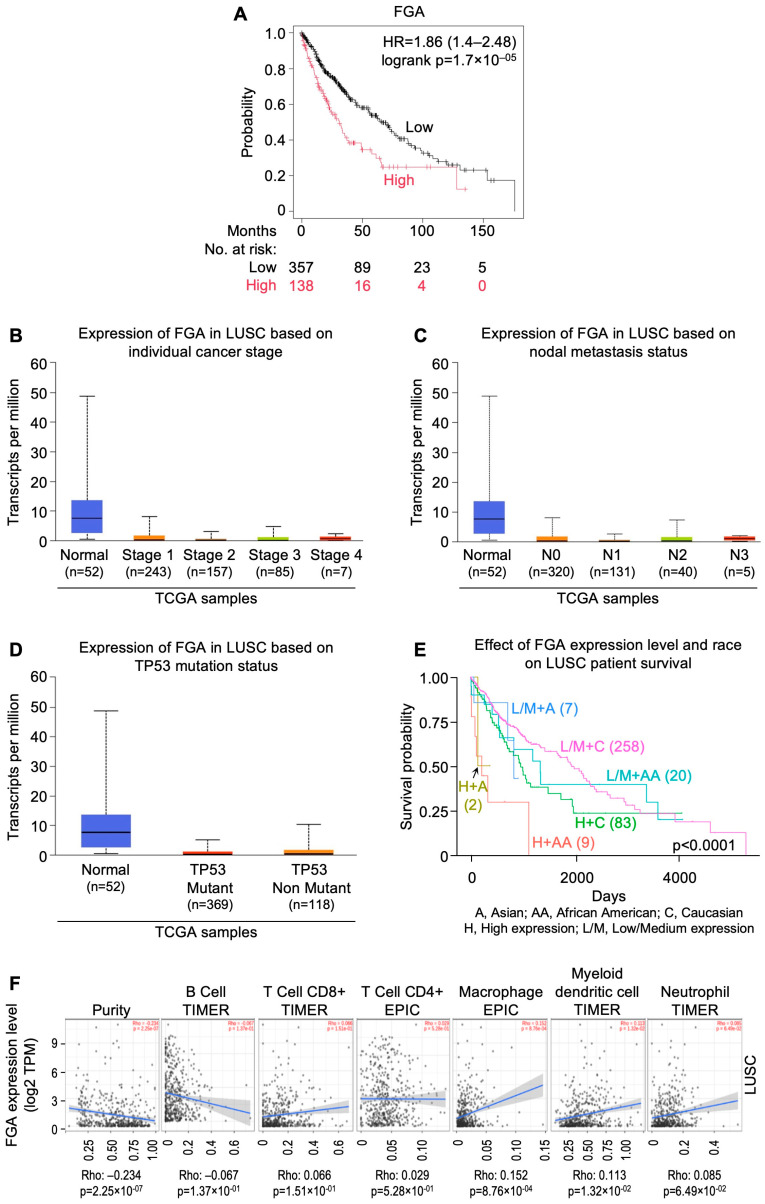
*FGA* gene expression as a candidate prognostic indicator for lung squamous carcinomas from UALCAN-based analysis of TCGA data. (**A**) Relation between gene expression and overall survival as depicted by KM Plotter. (**B**) Relation between gene expression and disease stage. (**C**) Relation between gene expression and metastatic status. (**D**) Relation between gene expression and *TP53* mutation status. (**E**) Relation between gene expression, ethnicity, and overall survival. (**F**) Correlation of gene expression with tumor infiltrating lymphocytes using TIMER 2.0. [LUSC: lung squamous carcinomas; NSCLC: non-small cell lung carcinoma; TCGA: The Cancer Genome Atlas; UALCAN: University of Alabama at Birmingham CANcer data analysis Portal].

**Figure 6:**
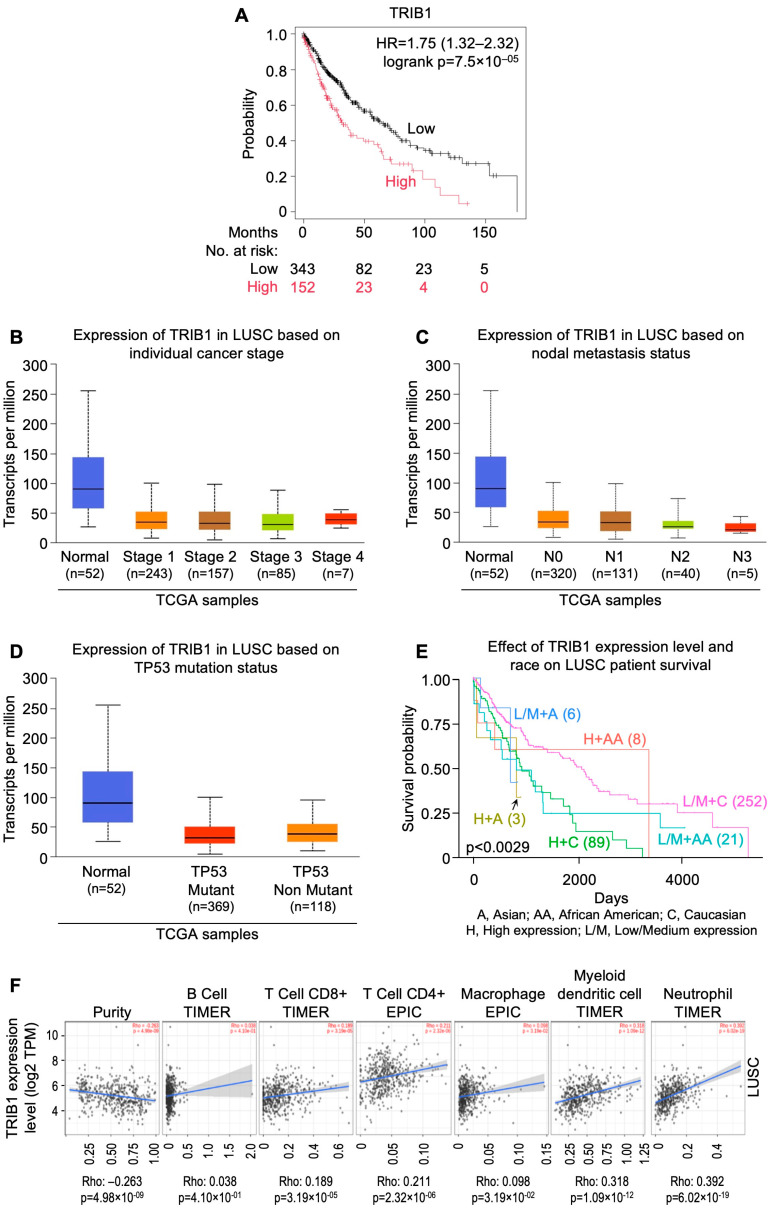
*TRIB1* gene expression as a candidate prognostic indicator for lung squamous carcinomas from UALCAN-based analysis of TCGA data. (**A**) Relation between gene expression and overall survival as depicted by KM Plotter. (**B**) Relation between gene expression and disease stage. (**C**) Relation between gene expression and metastatic status. (**D**) Relation between gene expression and *TP53* mutation status. (**E**) Relation between gene expression, ethnicity, and overall survival. (**F**) Correlation of gene expression with tumor infiltrating lymphocytes using TIMER 2.0. [LUSC: lung squamous carcinomas; NSCLC: non-small cell lung carcinoma; TCGA: The Cancer Genome Atlas; UALCAN: University of Alabama at Birmingham CANcer data analysis Portal].

We performed in silico analysis for candidate prognostic markers from the LUAD and LUSC cohorts of TCGA by using the UALCAN data analysis portal [64,65]. This uncovered a strong association between overexpression of *MYO1E* (HR = 2.3; CI: 1.7–3.12; p_log-rank_ = 2.9 × 10^−8^), *FAM83* homolog (HR = 2.43; CI: 1.78–3.31; p_log-rank_ = 6.7 × 10^−9^), and *DKK1* (HR = 2.55; CI: 1.9–3.42; p_log-rank_ = 1.2 × 10^−10^), and poor prognosis in LUAD (Figure 2A, Figure 3A and Figure 4A). In addition, expression of *FGA* (HR=1.86; CI: 1.4–2.48; p_log-rank_ = 1.7 × 10^−5^) and *TRIB1* (HR=1.75; CI: 1.32–2.32; p_log-rank_ = 7.5 × 10^−5^) were found to be strongly correlated with poor prognosis in LUSC (Figure 5A and Figure 6A). *FAM83* homologs showed the second highest expression in lung adenocarcinoma, which was also corroborated with immunohistochemistry data from The Human Protein Atlas (https://www.proteinatlas.org; accessed on 29 August 2024) linked to UALCAN (Supplementary Figure S1). In addition, overexpression of *MYO1E*, *FAM83*, and *DKK1* were distinctly related to increased pathological stage, nodal involvement, and *TP53* mutation in LUAD (Figure 2B–D, Figure 3B–D and Figure 4B–D). However, such a relation was only mildly observed for *FGA* and *TRIB1* in LUSC (Figure 5B–D and Figure 6B–D). Prognosis associated with the expression levels of all five candidate prognostic markers were found to significantly vary amongst different ethnicities, including African American, Asian, and Caucasian (Figure 2E, Figure 3E, Figure 4E, Figure 5E and Figure 6E).

An earlier insilico study has suggested that the FAM83 family carries prognostic significance in NSCLC [110]. FAM83 functions through ERK and PI3K/mTOR/AKT pathways and drives disease progression through WNT and HIPPO pathways [111,112]. The long non-coding antisense RNA, FAM83A-AS1, was found to upregulate FAM83A mRNA, thereby promoting NSCLC [113]. On the other hand, miRNA-1 inhibits *FAM83A*, associating the combination of low miR-1-3p and high FAM83A with poor survival in adenocarcinoma [114]. In addition, MYO1E, which functions through the MAPK pathway, was found to be significantly overexpressed among advanced stages of LUAD, but not LUSC, owing to its low level of methylation [115]. Overexpression of *DKK1* reduced median survival in NSCLC patients, suggesting its prognostic significance [116]. DKK1 could also promote epithelial-to-mesenchymal transition through the β-catenin pathway [117] and has been reported to be correlated with cisplatin resistance [118]. Further, *TRIB1* overexpression correlated with poor overall survival and multi-drug resistance, and was found in cisplatin-pretreated NSCLC, dependent on CEBPB regulation [119]. Increased expression and secretion of FGA from hepatocytes were found to be correlated with TKI resistance [120]. Relevant details regarding these markers are provided in Table 2.

## 9. Further Clues from the Cancer Genome Atlas (TCGA) and cBioPortal Analyzing Clinically Relevant Parameters Using TIMER, KM PLOTTER, and DepMap

The role of tumor infiltrating lymphocytes (TILs) is crucial for predicting tumor growth, progression, and response to the therapies [121]. It was observed that *MYO1E*, *FAM83A*, and *DKK1* showed a negative correlation with B cells (Figure 2F, Figure 3F and Figure 4F), whereas *FAM83A* showed a negative correlation with maximum TILs (Figure 3F). The results depicted that high expression of these genes may be associated with poor prognosis in patients with lung adenocarcinoma. On the other hand, TRIB1 has positively correlated with tumor infiltrating lymphocytes (Figure 5F), and *FGA* showed no significant correlation with TILs (Figure 6F).

**Figure 7:**
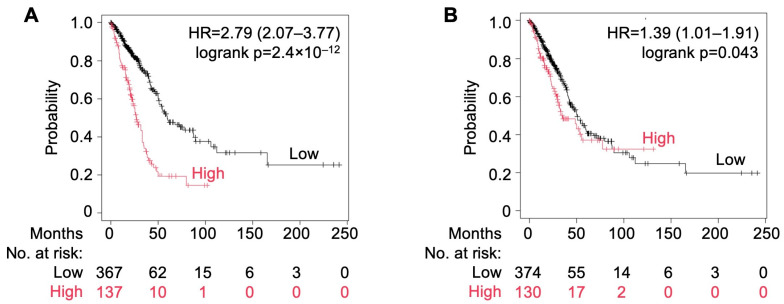
(**A**) Overall survival using multivariate analysis of *MYO1E*, *FAM83A*, and *DKK1* expression in lung adenocarcinoma (LUAD) patients. (**B**) Overall survival using multivariate analysis of *FGA* and *TRIB1* expression in lung squamous carcinoma (LUSC) patients using KM Plotter.

Furthermore, analysis through KM Plotter showed that the cumulative (HR = 2.79; CI: 2.07–3.77; p_log-rank_ = 2.4 × 10^−12^) high expression of *MYO1E*, *FAM83A*, and *DKK1* also led to poor survival in patients of lung adenocarcinoma (Figure 7A), while that of *FGA* and *TRIB1* (HR = 1.39; CI: 1.01–1.91; p_log-rank_ = 0.043) affected the survival of patients with lung squamous carcinoma (Figure 7B).

**Figure 8:**
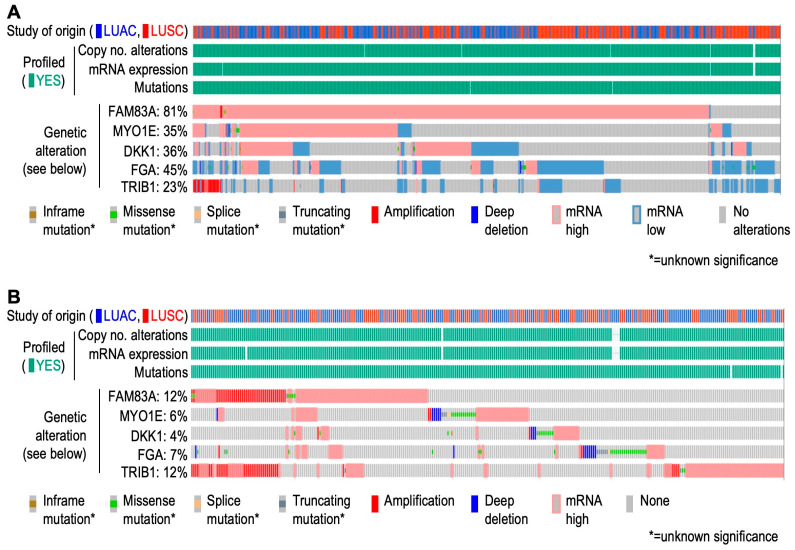
Oncoprint: cBioPortal-based view of genomic alterations including gene expression in NSCLC cohorts from TCGA. (**A**) Gene expression is compared in reference to adjacent normal tissues. (**B**) Gene expression is compared about diploid tumors. [NSCLC: non-small cell lung carcinoma; TCGA: The Cancer Genome Atlas; LUAC: lung adenocarcinoma; LUSC: lung squamous carcinoma].

**Figure 9:**
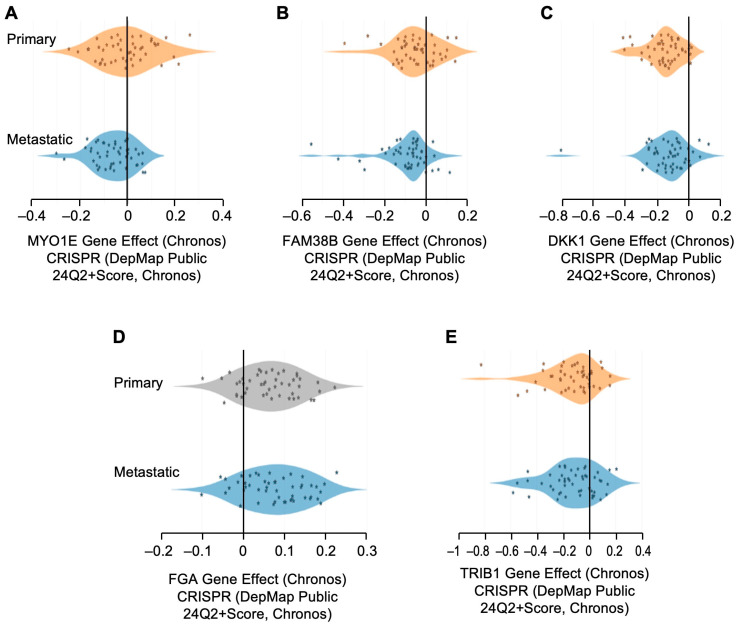
DepMap analysis of gene effect of (**A**) *MYO1E*, (**B**) *FAM83A*, (**C**) *DKK1*, (**D**) *FGA*, and (**E**) *TRIB1* using the Chronos model on different cell lines of non-small cell lung carcinoma (NSCLC).

We further studied genomic alteration in NSCLC from The Cancer Genome Atlas (TCGA), as well as other non-TCGA data using cBioPortal (https://www.cbioportal.org/; accessed on 29 August 2024). Genetic alterations found in *FAM83A* (81%), *MYO1E* (35%), *DKK1* (36%), *FGA* (45%), and TRIB1 (23%) in tumors mainly comprised changes in mRNA expressions compared to adjacent normal tissues (Figure 8A). When compared with diploid tumors, genetic alterations were predominated by overexpression of these genes (Figure 8B). Overexpression of *FAM83A* and *TRIB1* was found to be supported by frequent copy number amplifications (Figure 8B). Analyses of genetic alterations from non-TCGA studies also depicted frequent copy number amplifications of *FAM83A* and *TRIB1*, in absence of available expression data (Supplementary Figure S2). We further explored the DepMap portal to understand the gene effect on NSCLC proliferation and found that the loss of gene function of *MYO1E*, *FAM83A*, *DKK1*, *FGA*, and *TRIB1* showed a negative effect on maximum cell lines of primary and metastatic origin for NSCLC (Figure 9). Available clinical data on TCGA and non-TCGA cohorts revealed that the majority of the patients from both cohorts did not receive neo-adjuvant therapy, but were treated with adjuvant radiotherapy (Table 3). However, data on adjuvant treatment were grossly unavailable for the non-TCGA cohorts.

## 10. Discussion

Successful treatment of a heterogeneous disease like NSCLC substantially depends on the categorization of patient subsets based on prognostic and predictive biomarkers, thereby supporting different clinical outcomes and therapy regimens. Prognostic biomarkers indicate the chances of clinical outcomes in terms of overall/progression-free/disease-free survival, irrespective of specific therapeutics, and can reduce over- or under-treatment of patients and increase their quality of life.

To the best of our knowledge, there are no clinically validated prognostic biomarkers for NSCLC, yet. Therefore, we reviewed the ongoing preclinical research on prognostic biomarkers separately for the LUAD and LUSC subtypes of NSCLC. In this context, we found five more candidate prognostic biomarkers for NSCLC by data-mining from UALCAN and cBioPortal. These included *MYO1E*, *FAM83A*, and *DKK1* for LUAD, and *FGA* and *TRIB1* for LUSC. Higher expression of these five biomarkers showed poor prognosis.

Contrary to many of the preclinical prognostic biomarkers reviewed here that only show statistical power when combined with others, the five new biomarkers carry individual statistical significance in the prognostication of NSCLC. The majority of these five candidate biomarkers had been previously implicated in NSCLC with a focus on ICI resistance in various recent basic research studies. As an example, FAM83 family proteins are associated with resistance against tyrosine kinase inhibitors (TKIs), thereby becoming potential prognostic biomarkers of therapeutic resistance in cancer [122,123]. FAM83D was found to be associated with immunomodulation in hepatocellular carcinoma [124]. FAM83 family proteins were also overexpressed among immune-infiltrative subtypes of various cancers, suggesting that they merit further studies in the context of immune therapy resistance [122]. The prognostic significance of FAM83 family proteins has also been suggested in breast cancer [125]. In a previous study, decreased DNA methylation and high RNA expression of *MYO1E* were found to portray a lower median survival in LUAD but not LUSC patients [115]. MYO1E was also reported to be associated with PD-1/PD-L1 overexpression in ovarian cancers showing immunotherapeutic implications [126]. DKK1 is associated with an immune-suppressive microenvironment of various cancers, making it a potential indicator of immune-therapeutic resistance [127]. DKK1 expression together with CTNNB1 was found to worsen overall survival, as well as relapse-free survival, even in the presence of CD8+ tumor-infiltrating lymphocytes in biliary tract cancer [128]. On the other hand, *TRIB1* was found to be overexpressed by tumor-associated macrophages in breast cancer, and eventually reduced T cell infiltration by inhibiting IL-15, having immune-resistant underpinnings [129]. TRIB1 has also been reviewed to be associated with drugresistance in various cancers [130].

A rich repertoire of prognostic biomarkers and knowledge about their crosstalk with existing therapeutic interventions will help to address the heterogeneity in NSCLC and facilitate precision therapy. Development and validation of genetic testing-based prognostic biomarkers may appear expensive, but they can save the patients from the adverse effects and monetary expenditure of unnecessary treatments, and further guide them to relevant therapies. The use of artificial intelligence (AI) in healthcare has led to a steadily increasing amount of research, suggesting that AI technology may enhance the ability to predict the effectiveness of immunotherapy, thus enabling precision medicine. AI has the potential to play a substantial role in comprehending and tackling this resistance by using diverse data-specific methodologies, such as genomics, radiomics, proteomics, and histopathological features [131,132].

## 11. Conclusions

A search for prognostic biomarkers for NSCLC has remained a gray zone, lacking predictive accuracy, and demanding more research to meet the challenge of precision treatment. This may help reduce over- or under-treatment by tracking therapeutic effects with precise prognostic indicators. Our study revealed that overexpression of five genes serves as prospective prognostic indicators/biomarkers for NSCLC. Three of these genes (*MYO1E*, *FAM83A*, and *DKK1*) were LUAD-related, and two genes (*FGA* and *TRIB1*) were LUSC-related. This finding was corroborated by higher expression of the genes, along with copy number amplification of *FAM83* and *TRIB1*. Thus, unlike most studies that have reported prognostic indicators for LUAD only, we identified distinct biomarkers in LUAD as well as LUSC. Further support for these findings can be gained from validation in diverse cohorts of cancer patients in preclinical experiments and clinical trials. The novel candidates increase the prospects of accurate prognostics required to fight new challenges of therapy resistance in NSCLC and improve patient survival.

## Figures and Tables

**Table 1:** Most frequent genomic alterations in NSCLC.

Gene	Alteration Type	Observed Frequency *
*KRAS*	Mutation	15–25%
*EGFR*	Mutation	11–35%
*FGFR1*	Amplification	20%
*ALK*	Rearrangement	3–7%
*HER2*	Mutation, Amplification	2–4%
*MET*	Amplification	2–4%

* Genomic alterations occurring at a frequency < 2% include (but are not limited to) other genes like *BRAF*, *PIK3CA*, *AKT1*, *MEK1*, *NRAS*, *RET*, *RB1*, and *NTRK*.

**Table 2:** Previous reports on candidate prognostic biomarkers.

Candidate Prognostic Biomarker	Alteration	Mechanism	Histology	Phenotype or Functions ^‡^	Reference
*MYO1E*	Overexpression	MAPK pathway	NSCLC adenocarcinoma	intracellular movement and membrane trafficking	[115]
*FAM83* family	Overexpression	ERK pathway; PI3K/mTOR/AKT	NSCLC adenocarcinoma	protein kinase binding activity	[110,111,112,113,114]
*DKK1*	Overexpression	CTNNB1 pathway	NSCLC adenocarcinoma	role in embryonic development	[116,117,118]
*FGA*	Overexpression	Secretory protein	NSCLC squamous carcinoma	subunit of the coagulation factor fibrinogen	[120]
*TRIB1*	Overexpression	MAPK pathway	NSCLC squamous carcinoma	regulation of protein kinase activity	[119]

^‡^ Searched from Genecards (https://www.genecards.org/) (accessed on 29 August 2024).

**Table 3:** Treatment received by patients from TCGA and non-TCGA studies included in the review.

	**Neoadjuvant Treatment Data** (available for 514 out of 566 samples of the cohort)	**Adjuvant Radiation Therapy Data** (available for 467 out of 566 samples of the cohort)
**TCGA Study on Lung Adenocarcinoma**	treated = 3/514 (0.58%)	treated = 61/467 (13.06%)
	untreated = 511/514 (99.42%)	untreated = 406/467 (86.94%)
	**Neoadjuvant Treatment Data** (available for 485 out of 487 samples of the cohort)	**Adjuvant Radiation Therapy Data** (available for 422 out of 487 samples of the cohort)
**TCGA Study on Lung Squamous Cell Carcinoma**	treated = 5/485 (1.03%)	treated = 52/422 (12.32%)
	untreated = 480/485 (98.97%)	untreated = 370/422 (87.68%)
	**Prior Treatment Data** (available for 6 out of 186 samples of the cohort)	**Adjuvant Radiation Therapy Data** (available for 8 out of 186 samples of the cohort)
Non-TCGA Study on NSCLC * (Lung Adenocarcinoma, MSKCC, 2021)	treated = 0/6 (0.00%)	treated = 2/8 (25.0%)
	untreated = 6/6 (100%)	untreated = 6/8 (75.0%)
	**Prior Treatment Data** (available for 2591 out of 2621 samples of the cohort)	**Adjuvant Radiation Therapy Data** (not available)
Non-TCGA Study on NSCLC * (Metastatic Non-Small Cell Lung Cancer (MSKCC, 2022)	treated = 1030/2591 (39.75%)	NA
	untreated = 1561/2591 (60.25%)	

* Treatment data are available for only these two non-TCGA NSCLC studies in cBioPortal; NA = not applicable. MSKCC, Memorial Sloan Kettering Cancer Center.

## Data Availability

Not applicable.

## Supplementary Materials

The following supporting information can be downloaded at: https://www.mdpi.com/article/10.3390/cells13211785/s1, Figure S1: Over-expression of FAM83 homologs in NSCLC; Figure S2: Oncoprint: cBioPortal based view of Genomic alterations in NSCLC from non-TCGA cohorts showing amplifications in FAM83A and TRIB1.

## List of Abbreviations

*AKT1*	AKT Serine/Threonine Kinase 1;
*ALK*	anaplastic lymphoma kinase;
Amp	amplifications;
*APCs*	antigen-presenting cells;
*BRAF*	B-Raf proto-oncogene, serine/threonine kinase;
circ_001569	circular RNA;
CTL	cytotoxic T-cell;
*CTLA-4*	cytotoxic T-lymphocyte-associated protein 4;
*DKK1*	Dickkopf-related protein 1;
ECOG	Eastern Cooperative Oncology Group;
*EGFR*	Epidermal growth factor receptor;
*FAM83*	Family with Sequence Similarity 83;
*FGA*	fibrinogen alpha chain;
*FGFR1*	fibroblast growth factor receptor;
*HER2*	human epidermal growth factor receptor 2;
*HLA*	human leukocyte antigen;
ICIs	immune checkpoint inhibitors;
*IFN*	interferon;
*JAK/STAT*	Janus kinase and signal transducer and activator of transcription;
*KRAS*	Kirsten rat sarcoma viral oncogene homologue;
LINC00152	(Non-coding RNA-Cytor or Cytoskeleton Regulator RNA);
LUAD	lung adenocarcinomas;
*MEK1*	mitogen-activated protein kinase kinase;
Mut	mutation;
*MYO1E*	non-muscle class I myosin protein;
*MKI67*	Antigen Kiel 67, marker of proliferation;
KPS	Karnofsky performance status;
*MET*	MNNG HOS transforming gene;
NOS	not otherwise specified;
*NRAS*	neuro rearranged during transfection blastoma RAS viral oncogene homolog;
NSCLC	non-small cell lung cancer;
*NTRK*	neurotrophic tyrosine receptor kinase;
*PD-1*	programmed cell death protein 1;
*PD-L1*	programmed cell death ligand 1;
*PIK3CA*	phosphatidylinositol-4,5-bisphosphate 3-kinase catalytic subunit alpha;
*PINK1*	PTEN induced kinase 1;
*RB1*	retinoblastoma 1;
Rearr	rearrangements;
*RET*	rearranged during transfection;
*ROS1*	v-ros avian UR2 sarcoma virus oncogene homolog 1;
SCLC	small cell lung cancer;
*Src*	v-src sarcoma (Schmidt-Ruppin A-2) viral oncogene homolog
SCT	small cell transformation;
*TCR*	T-cell receptor;
TCGA	The Cancer Genome Atlas;
TILs	tumor-infiltrating lymphocytes;
TIME	tumor immune microenvironment;
TKI	tyrosine kinase inhibitor;
TNM	tumor (T), extent of spread to the lymph nodes (N), and presence of metastasis (M);
*TRIB1*	tribbles pseudokinase 1;
UALCAN	University of Alabama at Birmingham CANcer (UALCAN).

## References

[1] Bray F., Laversanne M., Sung H., Ferlay J., Siegel R.L., Soerjomataram I., Jemal A. (2024). Global cancer statistics 2022: GLOBOCAN estimates of incidence and mortality worldwide for 36 cancers in 185 countries. CA Cancer J. Clin..

[2] Schabath M.B., Cote M.L. (2019). Cancer Progress and Priorities: Lung Cancer. Cancer Epidemiol. Biomark. Prev..

[3] Guo Q., Liu L., Chen Z., Fan Y., Zhou Y., Yuan Z., Zhang W. (2022). Current treatments for non-small cell lung cancer. Front. Oncol..

[4] Hiltbrunner S., Cords L., Kasser S., Freiberger S.N., Kreutzer S., Toussaint N.C., Grob L., Opitz I., Messerli M., Zoche M. (2023). Acquired resistance to anti-PD1 therapy in patients with NSCLC associates with immunosuppressive T cell phenotype. Nat. Commun..

[5] Peng D., Liang P., Zhong C., Xu P., He Y., Luo Y., Wang X., Liu A., Zeng Z. (2022). Effect of EGFR amplification on the prognosis of EGFR-mutated advanced non–small-cell lung cancer patients: A prospective observational study. BMC Cancer.

[6] Shi Y., Au J.S.K., Thongprasert S., Srinivasan S., Tsai C.M., Khoa M.T., Heeroma K., Itoh Y., Cornelio G., Yang P.C. (2014). A prospective, molecular epidemiology study of EGFR mutations in Asian patients with advanced non-small-cell lung cancer of adenocarcinoma histology (PIONEER). J. Thorac. Oncol..

[7] Westover D., Zugazagoitia J., Cho B.C., Lovly C.M., Paz-Ares L. (2018). Mechanisms of acquired resistance to first- and second-generation EGFR tyrosine kinase inhibitors. Ann. Oncol..

[8] Panda G.S., Noronha V., Shah D., John G., Chougule A., Patil V., Kumar R., Menon N., Singh A., Chandrani P. (2022). Treatment pattern and outcomes in de novo T790M-mutated non-small cell lung cancer. Ecancermedicalscience.

[9] Ko B., Paucar D., Halmos B. (2017). EGFR T790M: Revealing the secrets of a gatekeeper. Lung Cancer Targets Ther..

[10] Bencze E., Bogos K., Kohánka A., Báthory-Fülöp L., Sárosi V., Csernák E., Bittner N., Melegh Z., Tóth E. (2022). EGFR T790M Mutation Detection in Patients with Non-Small Cell Lung Cancer After First Line EGFR TKI Therapy: Summary of Results in a Three-Year Period and a Comparison of Commercially Available Detection Kits. Pathol. Oncol. Res..

[11] Yang Z., Hackshaw A., Feng Q., Fu X., Zhang Y., Mao C., Tang J. (2017). Comparison of gefitinib, erlotinib and afatinib in non-small cell lung cancer: A meta-analysis. Int. J. Cancer.

[12] Daaboul N., Nicholas G., Laurie S.A. (2018). Algorithm for the treatment of advanced or metastatic squamous non-small-cell lung cancer: An evidence-based overview. Curr. Oncol..

[13] Wecker H., Waller C.F. (2018). Afatinib. Recent Results Cancer Res..

[14] Zheng D., Hu M., Bai Y., Zhu X., Lu X., Wu C., Wang J., Liu L., Wang Z., Ni J. (2017). EGFR G796D mutation mediates resistance to osimertinib. Oncotarget.

[15] Ramalingam S.S., Yang J.C.H., Lee C.K., Kurata T., Kim D.W., John T., Nogami N., Ohe Y., Mann H., Rukazenkov Y. (2018). Osimertinib as first-line treatment of EGFR mutation-positive advanced non-small-cell lung cancer. J. Clin. Oncol..

[16] Yamaura T., Muto S., Mine H., Takagi H., Watanabe M., Ozaki Y., Inoue T., Fukuhara M., Okabe N., Matsumura Y. (2020). Genetic alterations in epidermal growth factor receptor-tyrosine kinase inhibitor-naïve non-small cell lung carcinoma. Oncol. Lett..

[17] Gadgeel S., Horn L. (2019). Approach to Anaplastic Lymphoma Kinase (ALK) Gene Rearranged Non–Small Cell Lung Cancer (NSCLC). Pulmonary Adenocarcinoma: Approaches to Treatment.

[18] Forde P.M., Rudin C.M. (2012). Crizotinib in the treatment of non-small-cell lung cancer. Expert. Opin Pharmacother..

[19] Soda M., Choi Y.L., Enomoto M., Takada S., Yamashita Y., Ishikawa S., Fujiwara S., Watanabe H., Kurashina K., Hatanaka H. (2007). Identification of the transforming EML4-ALK fusion gene in non-small-cell lung cancer. Nature.

[20] Franco R., Rocco G., Marino F.Z., Pirozzi G., Normanno N., Morabito A., Sperlongano P., Stiuso P., Luce A., Botti G. (2013). Anaplastic lymphoma kinase: A glimmer of hope in lung cancer treatment?. Expert Rev. Anticancer Ther..

[21] Huang R.S.P., Haberberger J., Sokol E., Schrock A.B., Danziger N., Madison R., Trabucco S., Jin D., Pavlick D., Ramanan V. (2021). Clinicopathologic, genomic and protein expression characterization of 356 ROS1 fusion driven solid tumors cases. Int. J. Cancer.

[22] Cui M., Han Y., Li P., Zhang J., Ou Q., Tong X., Zhao R., Dong N., Wu X., Li W. (2020). Molecular and clinicopathological characteristics of ROS1-rearranged non-small-cell lung cancers identified by next-generation sequencing. Mol. Oncol..

[23] Zhu Y.C., Xu C.W., Ye X.Q., Yin M.X., Zhang J.X., Du K.Q., Zhang Z.H., Hu J. (2016). Lung cancer with concurrent EGFR mutation and ROS1 rearrangement: A case report and review of the literature. Onco. Targets Ther..

[24] Uguen A., Schick U., Quéré G. (2017). A Rare Case of ROS1 and ALK Double Rearranged Non-Small Cell Lung Cancer. J. Thorac. Oncol..

[25] Pathak N., Chitikela S., Malik P.S. (2021). Recent advances in lung cancer genomics: Application in targeted therapy. Adv. Genet..

[26] Rothschild S.I., Gautschi O. (2013). Crizotinib in the treatment of non-small-cell lung cancer. Clin. Lung Cancer.

[27] Šutić M., Vukić A., Baranašić J., Försti A., Džubur F., Samaržija M., Jakopović M., Brčić L., Knežević J. (2021). Diagnostic, Predictive, and Prognostic Biomarkers in Non-Small Cell Lung Cancer (NSCLC) Management. J. Pers. Med..

[28] Ahmadzada T., Kao S., Reid G., Boyer M., Mahar A., Cooper W.A. (2018). An Update on Predictive Biomarkers for Treatment Selection in Non-Small Cell Lung Cancer. J. Clin. Med..

[29] Khunger A., Khunger M., Velcheti V. (2018). Dabrafenib in combination with trametinib in the treatment of patients with BRAF V600-positive advanced or metastatic non-small cell lung cancer: Clinical evidence and experience. Ther. Adv. Respir. Dis..

[30] Wolf J., Seto T., Han J.-Y., Reguart N., Garon E.B., Groen H.J.M., Tan D.S.-H., Hida T., De Jonge M.J., Orlov S.V. (2019). Capmatinib (INC280) in METΔex14-mutated advanced non-small cell lung cancer (NSCLC): Efficacy data from the phase II GEOMETRY mono-1 study. J. Clin. Oncol..

[31] Paik P.K., Felip E., Veillon R., Paik P.K., Felip E., Veillon R., Sakai H., Cortot A.B., Garassino M.C., Mazieres J. (2020). Tepotinib in Non-Small-Cell Lung Cancer with MET Exon 14 Skipping Mutations. N. Engl. J. Med..

[32] Drusbosky L.M., Rodriguez E., Dawar R., Ikpeazu C.V. (2021). Therapeutic strategies in RET gene rearranged non-small cell lung cancer. J. Hematol. Oncol..

[33] Bendell J.C., Lim K.-H., Burkard M.E., Klempner S.J., Socinski M.A., Gadgeel S.M., Reckamp K.L., Leland S.M., Plessinger D., Kunkel L.A. (2021). CRESTONE: Clinical study of response to seribantumab in tumors with neuregulin-1 (NRG1) fusions—A phase II study of the anti-HER3 mAb for advanced or metastatic solid tumors (NCT04383210). J. Clin. Oncol..

[34] Dingemans A.-M.C., Smit E.F., De Langen J., van Tinteren H. (2019). Chemotherapy in KRAS-mutated chemotherapy naive non-small cell lung cancer patients: A phase III comparing ci.;platin-pemetrexed with carboplatin-paclitaxel-bevacizumab: NVALT 22 (NCT02743923). J. Clin. Oncol..

[35] Li B.T., Smit E.F., Goto Y., Nakagawa K., Udagawa H., Mazières J., Nagasaka M., Bazhenova L., Saltos A.N., Felip E. (2022). Trastuzumab Deruxtecan in HER2-Mutant Non-Small-Cell Lung Cancer. N. Engl. J. Med..

[36] Smit E.F., Peters S., Dziadziuszko R., Dafni U., Wolf J., Wasąg B., Biernat W., Finn S., Kammler R., Tsourti Z. (2017). A single-arm phase II trial of afatinib in pretreated patients with advanced NSCLC harboring a HER2 mutation: The ETOP NICHE trial. J. Clin. Oncol..

[37] Chai X., Zhang X., Li W., Chai J. (2021). Small cell lung cancer transformation during antitumor therapies: A systematic review. Open Med..

[38] Xu J., Xu L., Wang B., Kong W., Chen Y., Yu Z. (2022). Outcomes in Patients with Lung Adenocarcinoma with Transformation to Small Cell Lung Cancer After EGFR Tyrosine Kinase Inhibitors Resistance: A Systematic Review and Pooled Analysis. Front. Oncol..

[39] Zhang C., Lin L., Guo X., Chen P. (2020). Significance of genetic sequencing in patients with lung adenocarcinoma with transformation to small cell lung cancer: A case report and systematic review. Transl. Cancer Res..

[40] Shaurova T., Zhang L., Goodrich D.W., Hershberger P.A. (2020). Understanding Lineage Plasticity as a Path to Targeted Therapy Failure in EGFR-Mutant Non-small Cell Lung Cancer. Front. Genet..

[41] Topalian S.L., Hodi F.S., Brahmer J.R., Gettinger S.N., Smith D.C., McDermott D.F., Powderly J.D., Sosman J.A., Atkins M.B., Leming P.D. (2019). Five-Year Survival and Correlates Among Patients with Advanced Melanoma, Renal Cell Carcinoma, or Non-Small Cell Lung Cancer Treated With Nivolumab. JAMA Oncol..

[42] Siciliano M.A., Caridà G., Ciliberto D., d’Apolito M., Pelaia C., Caracciolo D., Riillo C., Correale P., Galvano A., Russo A. (2022). Efficacy and safety of first-line checkpoint inhibitors-based treatments for non-oncogene-addicted non-small-cell lung cancer: A systematic review and meta-analysis. ESMO Open.

[43] Guaitoli G., Tiseo M., Di Maio M., Friboulet L., Facchinetti F. (2021). Immune checkpoint inhibitors in oncogene-addicted non-small cell lung cancer: A systematic review and meta-analysis. Transl. Lung Cancer Res..

[44] Clarke J.M., George D.J., Lisi S., Salama A.K.S. (2018). Immune Checkpoint Blockade: The New Frontier in Cancer Treatment. Target. Oncol..

[45] Zhou K., Li S., Zhao Y., Cheng K. (2023). Mechanisms of drug resistance to immune checkpoint inhibitors in non-small cell lung cancer. Front. Immunol..

[46] Liu Y., Liu Z., Yang Y., Cui J., Sun J., Liu Y. (2023). The prognostic and biology of tumour-infiltrating lymphocytes in the immunotherapy of cancer. Br. J. Cancer.

[47] Presti D., Dall’Olio F.G., Besse B., Ribeiro J.M., Di Meglio A., Soldato D. (2022). Tumor infiltrating lymphocytes (TILs) as a predictive biomarker of response to checkpoint blockers in solid tumors: A systematic review. Crit. Rev. Oncol. Hematol..

[48] Zhang Y., Chen L. (2016). Classification of Advanced Human Cancers Based on Tumor Immunity in the MicroEnvironment (TIME) for Cancer Immunotherapy. JAMA Oncol..

[49] Vesely M.D., Zhang T., Chen L. (2022). Resistance Mechanisms to Anti-PD Cancer Immunotherapy. Annu. Rev. Immunol..

[50] Vari F., Arpon D., Keane C., Hertzberg M.S., Talaulikar D., Jain S., Cui Q., Han E., Tobin J., Bird R. (2018). Immune evasion via PD-1/PD-L1 on NK cells and monocyte/macrophages is more prominent in Hodgkin lymphoma than DLBCL. Blood.

[51] Hinterleitner C., Strähle J., Malenke E., Hinterleitner M., Henning M., Seehawer M., Bilich T., Heitmann J., Lutz M., Mattern S. (2021). Platelet PD-L1 reflects collective intratumoral PD-L1 expression and predicts immunotherapy response in non-small cell lung cancer. Nat. Commun..

[52] Negrao M.V., Skoulidis F., Montesion M., Schulze K., Bara I., Shen V., Xu H., Hu S., Sui D., Elamin Y.Y. (2021). Oncogene-specific differences in tumor mutational burden, PD-L1 expression, and outcomes from immunotherapy in non-small cell lung cancer. J. Immunother. Cancer.

[53] Gong B., Kiyotani K., Sakata S., Nagano S., Kumehara S., Baba S., Besse B., Yanagitani N., Friboulet L., Nishio M. (2019). Secreted PD-L1 variants mediate resistance to PD-L1 blockade therapy in non-small cell lung cancer. J. Exp. Med..

[54] Mahoney K.M., Ross-Macdonald P., Yuan L., Song L., Veras E., Wind-Rotolo M., Mcdermott D.F., Stephen Hodi F., Choueiri T.K., Freeman G.J. (2022). Original research: Soluble PD-L1 as an early marker of progressive disease on nivolumab. J. Immunother. Cancer.

[55] Gettinger S., Choi J., Hastings K., Truini A., Datar I., Sowell R., Wurtz A., Dong W., Cai G., Melnick M.A. (2017). Impaired HLA Class I Antigen Processing and Presentation as a Mechanism of Acquired Resistance to Immune Checkpoint Inhibitors in Lung Cancer. Cancer Discov..

[56] Carbotti G., Nikpoor A.R., Vacca P., Gangemi R., Giordano C., Campelli F., Ferrini S., Fabbi M. (2017). IL-27 mediates HLA class I up-regulation, which can be inhibited by the IL-6 pathway, in HLA-deficient Small Cell Lung Cancer cells. J. Exp. Clin. Cancer Res..

[57] Correale P., Saladino R.E., Giannarelli D., Giannicola R., Agostino R., Staropoli N., Strangio A., Del Giudice T., Nardone V., Altomonte M. (2020). Distinctive germline expression of class I human leukocyte antigen (HLA) alleles and DRB1 heterozygosis predict the outcome of patients with non-small cell lung cancer receiving PD-1/PD-L1 immune checkpoint blockade. J. Immunother. Cancer.

[58] Schöffski P., Tan D.S.W., Martín M., Ochoa-de-Olza M., Sarantopoulos J., Carvajal R.D., Kyi C., Esaki T., Prawira A., Akerley W. (2022). Phase I/II study of the LAG-3 inhibitor ieramilimab (LAG525) ± anti-PD-1 spartalizumab (PDR001) in patients with advanced malignancies. J. Immunother. Cancer.

[59] Anderson A.C., Joller N., Kuchroo V.K. (2016). Lag-3, Tim-3, and TIGIT: Co-inhibitory Receptors with Specialized Functions in Immune Regulation. Immunity.

[60] Maruhashi T., Sugiura D., Okazaki I.M., Shimizu K., Maeda T.K., Ikubo J., Yoshikawa H., Maenaka K., Ishimaru N., Kosako H. (2022). Binding of LAG-3 to stable peptide-MHC class II limits T cell function and suppresses autoimmunity and anti-cancer immunity. Immunity.

[61] Morandi F., Fainardi E., Rizzo R., Rouas-Freiss N. (2014). The role of HLA-class Ib molecules in immune-related diseases, tumors, and infections. J. Immunol. Res..

[62] Shin D.S., Zaretsky J.M., Escuin-Ordinas H., Garcia-Diaz A., Hu-Lieskovan S., Kalbasi A., Grasso C.S., Hugo W., Sandoval S., Torrejon D.Y. (2017). Primary Resistance to PD-1 Blockade Mediated by JAK1/2 Mutations. Cancer Discov..

[63] Benci J.L., Xu B., Qiu Y., Wu T.J., Dada H., Twyman-Saint Victor C., Cucolo L., Lee D.S.M., Pauken K.E., Huang A.C. (2016). Tumor Interferon Signaling Regulates a Multigenic Resistance Program to Immune Checkpoint Blockade. Cell.

[64] Chandrashekar D.S., Karthikeyan S.K., Korla P.K., Patel H., Shovon A.R., Athar M., Netto G.J., Qin Z.S., Kumar S., Manne U. (2022). UALCAN: An update to the integrated cancer data analysis platform. Neoplasia.

[65] Chandrashekar D.S., Bashel B., Balasubramanya S.A.H., Creighton C.J., Ponce-Rodriguez I., Chakravarthi B.V.S.K., Varambally S. (2017). UALCAN: A Portal for Facilitating Tumor Subgroup Gene Expression and Survival Analyses. Neoplasia.

[66] Standfield L., Weston A.R., Barraclough H., Van Kooten M., Pavlakis N. (2011). Histology as a treatment effect modifier in advanced non-small cell lung cancer: A systematic review of the evidence. Respirology.

[67] Moosavi F., Giovannetti E., Saso L., Firuzi O. (2019). HGF/MET pathway aberrations as diagnostic, prognostic, and predictive biomarkers in human cancers. Crit. Rev. Clin. Lab. Sci..

[68] Furrer D., Paquet C., Jacob S., Diorio C., Furrer D., Paquet C., Jacob S., Diorio C., Lemamy G.-J. (2018). The Human Epidermal Growth Factor Receptor 2 (HER2) as a Prognostic and Predictive Biomarker: Molecular Insights into HER2 Activation and Diagnostic Implications. Cancer Prognosis.

[69] Lindeman N.I., Cagle P.T., Aisner D.L., Arcila M.E., Beasley M.B., Bernicker E.H., Colasacco C., Dacic S., Hirsch F.R., Kerr K. (2018). Updated molecular testing guideline for the selection of lung cancer patients for treatment with targeted tyrosine kinase inhibitors: Guideline from theCollege of American Pathologists, the International Association for the Study of Lung Cancer, and the Association for Molecular Pathology. J. Mol. Diagn..

[70] Wu Y.L., Planchard D., Lu S., Wu Y.L., Planchard D., Lu S., Sun H., Yamamoto N., Kim D.W., Tan D.S.W. (2019). Pan-Asian adapted Clinical Practice Guidelines for the management of patients with metastatic non-small-cell lung cancer: A CSCO-ESMO initiative endorsed by JSMO, KSMO, MOS, SSO and TOS. Ann. Oncol..

[71] Román M., Baraibar I., López I., Nadal E., Rolfo C., Vicent S., Gil-Bazo I. (2018). KRAS oncogene in non-small cell lung cancer: Clinical perspectives on the treatment of an old target. Mol. Cancer..

[72] Morris T.A., Khoo C., Solomon B.J. (2019). Targeting ROS1 Rearrangements in Non-small Cell Lung cancer: Crizotinib and newer generation tyrosine kinase inhibitors. Drugs.

[73] Lin Q., Zhang H., Ding H., Qian J., Lizaso A., Lin J., Han-Zhang H., Xiang J., Li Y., Zhu H. (2019). The association between BRAF mutation class and clinical features in BRAF-mutant Chinese non-small cell lung cancer patients. J. Transl. Med..

[74] Johnson R.M.G., Dong H. (2017). Functional Expression of Programmed Death-Ligand 1 (B7-H1) by Immune Cells and Tumor Cells. Front. Immunol..

[75] Dong H., Strome S.E., Salomao D.R., Tamura H., Hirano F., Flies D.B., Roche P.C., Lu J., Zhu G., Tamada K. (2002). Tumor-associated B7-H1 promotes T-cell apoptosis: A potential mechanism of immune evasion. Nat. Med..

[76] Lu S. (2019). Development of treatment options for Chinese patients with advanced squamous cell lung cancer: Focus on afatinib. Onco Targets Ther..

[77] Rosas G., Ruiz R., Araujo J.M., Pinto J.A., Mas L. (2019). ALK rearrangements: Biology, detection and opportunities of therapy in non-small cell lung cancer. Crit. Rev. Oncol. Hematol..

[78] Brustugun O.T., Khattak A.M., Trømborg A.K., Beigi M., Beiske K., Lund-Iversen M., Helland Å. (2014). BRAF-mutations in non-small cell lung cancer. Lung Cancer.

[79] Tissot C., Couraud S., Tanguy R., Bringuier P.P., Girard N., Souquet P.J. (2016). Clinical characteristics and outcome of patients with lung cancer harboring BRAF mutations. Lung Cancer.

[80] Bergethon K., Shaw A.T., Ou S.H., Katayama R., Lovly C.M., McDonald N.T., Massion P.P., Siwak-Tapp C., Gonzalez A., Fang R. (2012). ROS1 rearrangements define a unique molecular class of lung cancers. J. Clin. Oncol..

[81] Cai W., Su C., Li X., Fan L., Zheng L., Fei K., Zhou C. (2013). KIF5B-RET fusions in Chinese patients with non–small cell lung cancer. Cancer.

[82] Wang Y., Xu Y., Wang X., Sun C., Guo Y., Shao G., Yang Z., Qiu S., Ma K. (2019). RET fusion in advanced non-small-cell lung cancer and response to cabozantinib: A case report. Medicine.

[83] Gautschi O., Milia J., Filleron T., Wolf J., Carbone D.P., Owen D., Camidge R., Narayanan V., Doebele R.C., Besse B. (2017). Targeting RET in Patients with RET-Rearranged Lung Cancers: Results From the Global, Multicenter RET Registry. J. Clin. Oncol..

[84] Stransky N., Cerami E., Schalm S., Kim J.L., Lengauer C. (2014). The landscape of kinase fusions in cancer. Nat. Commun..

[85] Jonna S., Feldman R.A., Swensen J., Gatalica Z., Korn W.M., Borghaei H., Ma P.C., Nieva J.J., Spira A.I., Vanderwalde A.M. (2019). Detection of NRG1 Gene Fusions in Solid Tumors. Clin. Cancer Res..

[86] Pao W., Wang T.Y., Riely G.J., Miller V.A., Pan Q., Ladanyi M., Zakowski M.F., Heelan R.T., Kris M.G., Varmus H.E. (2005). KRAS mutations and primary resistance of lung adenocarcinomas to gefitinib or erlotinib. PLoS Med..

[87] Kim E.K., Kim K.A., Lee C.Y., Shim H.S. (2017). The frequency and clinical impact of HER2 alterations in lung adenocarcinoma. PLoS ONE.

[88] Mazières J., Peters S., Lepage B., Cortot A.B., Barlesi F., Beau-Faller M., Besse B., Blons H., Mansuet-Lupo A., Urban T. (2013). Lung cancer that harbors an HER2 mutation: Epidemiologic characteristics and therapeutic perspectives. J. Clin. Oncol..

[89] Detterbeck F.C., Boffa D.J., Kim A.W., Tanoue L.T. (2017). The Eighth Edition Lung Cancer Stage Classification. Chest.

[90] Prasad K.T., Kaur H., Muthu V., Aggarwal A.N., Behera D., Singh N. (2018). Interconversion of two commonly used performance tools: An analysis of 5844 paired assessments in 1501 lung cancer patients. World J. Clin. Oncol..

[91] Puderecki M., Szumiło J., Marzec-Kotarska B. (2020). Novel prognostic molecular markers in lung cancer. Oncol. Lett..

[92] Jin B.F., Yang F., Ying X.M., Gong L., Hu S.F., Zhao Q., Liao Y.D., Chen K.Z., Li T., Tai Y.H. (2018). Signaling protein signature predicts clinical outcome of non-small-cell lung cancer. BMC Cancer.

[93] Yang Z., Yang F., Zhang Y., Wang X., Shi J., Wei H., Sun F., Yu Y. (2018). Girdin protein: A potential metastasis predictor associated with prognosis in lung cancer. Exp. Ther. Med..

[94] Folescu R., Levai C.M., Grigoraş M.L., Arghirescu T.S., Talpoş I.C., Gîndac C.M., Zamfir C.L., Poroch V., Anghel M.D. (2018). Expression and significance of Ki-67 in lung cancer. Rom. J. Morphol. Embryol..

[95] Villa E., Proïcs E., Rubio-Patiño C., Obba S., Zunino B., Bossowski J.P., Rozier R.M., Chiche J., Mondragón L., Riley J.S. (2017). Parkin-Independent Mitophagy Controls Chemotherapeutic Response in Cancer Cells. Cell Rep..

[96] Feng C., Feng M., Gao Y., Zhao X., Peng C., Yang X., Zhang J. (2019). Clinicopathologic Significance of Intestinal-type Molecules’ Expression and Different EGFR Gene Status in Pulmonary Adenocarcinoma. Appl. Immunohistochem. Mol. Morphol..

[97] Zhao K., Li Z., Tian H. (2018). Twenty-gene-based prognostic model predicts lung adenocarcinoma survival. Onco Targets Ther..

[98] Xu F., Lin H., He P., He L., Chen J., Lin L., Chen Y. (2020). A TP53-associated gene signature for prediction of prognosis and therapeutic responses in lung squamous cell carcinoma. Oncoimmunology.

[99] Qin K., Hou H., Liang Y., Zhang X. (2020). Prognostic value of TP53 concurrent mutations for EGFR- TKIs and ALK-TKIs based targeted therapy in advanced non-small cell lung cancer: A meta-analysis. BMC Cancer.

[100] Gu J., Zhou Y., Huang L., Ou W., Wu J., Li S., Xu J., Feng J., Liu B. (2016). TP53 mutation is associated with a poor clinical outcome for non-small cell lung cancer: Evidence from a meta-analysis. Mol. Clin. Oncol..

[101] Ma X., Le Teuff G., Lacas B., Tsao M.S., Graziano S., Pignon J.P., Douillard J.Y., Le Chevalier T., Seymour L., Filipits M. (2016). Prognostic and predictive effect of TP53 mutations in patients with non-small cell lung cancer from adjuvant cisplatin-based therapy randomized trials: A LACE-bio pooled analysis. J. Thorac. Oncol..

[102] Ma Z., Wang Y., He B., Cui J., Zhang C., Wang H., Feng W., Wang B., Wei D., Wu Y. (2018). Expression of miR-590 in lung cancer and its correlation with prognosis. Oncol. Lett..

[103] Qin Y., Zhou X., Huang C., Li L., Liu H., Liang N., Chen Y., Ma D., Han Z., Xu X. (2018). Lower miR-340 expression predicts poor prognosis of non-small cell lung cancer and promotes cell proliferation by targeting CDK4. Gene.

[104] Zhu W.Y., Luo B., An J.Y., He J.Y., Chen D.D., Xu L.Y., Huang Y.Y., Liu X.G., Le H.B., Zhang Y.K. (2014). Differential Expression of miR-125a-5p and let-7e Predicts the Progression and Prognosis of Non-Small Cell Lung Cancer. Cancer Invest..

[105] Yanaihara N., Caplen N., Bowman E., Seike M., Kumamoto K., Yi M., Stephens R.M., Okamoto A., Yokota J., Tanaka T. (2006). Unique microRNA molecular profiles in lung cancer diagnosis and prognosis. Cancer Cell.

[106] Faversani A., Amatori S., Augello C., Colombo F., Porretti L., Fanelli M., Ferrero S., Palleschi A., Pelicci P.G., Belloni E. (2017). miR-494-3p is a novel tumor driver of lung carcinogenesis. Oncotarget.

[107] Ding L., Yao W., Lu J., Gong J.U.N., Zhang X. (2018). Upregulation of circ_001569 predicts poor prognosis and promotes cell proliferation in non-small cell lung cancer by regulating the Wnt/β-catenin pathway. Oncol. Lett..

[108] Feng S., Zhang J., Su W., Bai S., Xiao L., Chen X., Lin J., Reddy R.M., Chang A.C., Beer D.G. (2017). Overexpression of LINC00152 correlates with poor patient survival and knockdown impairs cell proliferation in lung cancer. Sci. Rep..

[109] Chen M., Liu B., Xiao J., Yang Y., Zhang Y. (2017). A novel seven-long non-coding RNA signature predicts survival in early stage lung adenocarcinoma. Oncotarget.

[110] Gan J., Li Y., Meng Q. (2020). Systematic Analysis of Expression Profiles and Prognostic Significance for FAM83 Family in Non-small-Cell Lung Cancer. Front. Mol. Biosci..

[111] Hu H., Wang F., Wang M., Liu Y., Wu H., Chen X., Lin Q. (2020). FAM83A is amplified and promotes tumorigenicity in non-small cell lung cancer via ERK and PI3K/Akt/mTOR pathways. Int. J. Med. Sci..

[112] Zheng Y.W., Li Z.H., Lei L., Liu C.C., Wang Z., Fei L.R., Yang M.Q., Huang W.J., Xu H.T. (2020). FAM83A Promotes Lung Cancer Progression by Regulating the Wnt and Hippo Signaling Pathways and Indicates Poor Prognosis. Front. Oncol..

[113] Shi R., Jiao Z., Yu A., Wang T. (2019). Long noncoding antisense RNA FAM83A-AS1 promotes lung cancer cell progression by increasing FAM83A. J. Cell Biochem..

[114] Liu P.J., Chen Y.H., Tsai K.W., Yeah H.Y., Yeh C.Y., Tu Y.T., Yang C.Y. (2020). Involvement of MicroRNA-1-FAM83A Axis Dysfunction in the Growth and Motility of Lung Cancer Cells. Int. J. Mol. Sci..

[115] Jusue-Torres I., Tiv R., Ricarte-Filho J.C., Mallisetty A., Contreras-Vargas L., Godoy-Calderon M.J., Khaddour K., Kennedy K., Valyi-Nagy K., David O. (2023). Myo1e overexpression in lung adenocarcinoma is associated with increased risk of mortality. Sci. Rep..

[116] Yamabuki T., Takano A., Hayama S., Ishikawa N., Kato T., Miyamoto M., Ito T., Ito H., Miyagi Y., Nakayama H. (2007). Dikkopf-1 as a Novel Serologic and Prognostic Biomarker for Lung and Esophageal Carcinomas. Cancer Res..

[117] Zhang J., Zhang X., Zhao X., Jiang M., Gu M., Wang Z., Yue W. (2017). DKK1 promotes migration and invasion of non-small cell lung cancer via β-catenin signaling pathway. Tumor Biol..

[118] Salim H., Zong D., Hååg P., Novak M., Mörk B., Lewensohn R., Lundholm L., Viktorsson K. (2015). DKK1 is a potential novel mediator of cisplatin-refractoriness in non-small cell lung cancer cell lines. BMC Cancer.

[119] Wang L., Liu X., Ren Y., Zhang J., Chen J., Zhou W., Guo W., Wang X., Chen H., Li M. (2017). Cisplatin-enriching cancer stem cells confer multidrug resistance in non-small cell lung cancer via enhancing TRIB1/HDAC activity. Cell Death Dis..

[120] Shang Z., Niu X., Zhang K., Qiao Z., Liu S., Jiang X., Cao C., Lu S., Xiao H. (2019). FGA isoform as an indicator of targeted therapy for EGFR mutated lung adenocarcinoma. J. Mol. Med..

[121] Brummel K., Eerkens A.L., de Bruyn M., Nijman H.W. (2023). Tumour-infiltrating lymphocytes: From prognosis to treatment selection. Br. J. Cancer.

[122] Yuan S., Huang Z., Qian X., Wang Y., Fang C., Chen R., Zhang X., Xiao Z., Wang Q., Yu B. (2022). Pan-cancer analysis of the FAM83 family and its association with prognosis and tumor microenvironment. Front. Genet..

[123] Bartel C.A., Parameswaran N., Cipriano R., Jackson M.W. (2016). FAM83 proteins: Fostering new interactions to drive oncogenic signaling and therapeutic resistance. Oncotarget.

[124] Meng T., Tong Z., Yang M.Y., Zhang Y., Liu Y., Wang Z.Z., Zhu L.X., Wu J. (2021). Immune implication of FAM83D gene in hepatocellular carcinoma. Bioengineered.

[125] Jin Y., Yu J., Jiang Y., Bu J., Zhu T., Gu X., Zhu X. (2022). Comprehensive analysis of the expression, prognostic significance, and function of FAM83 family members in breast cancer. World J. Surg. Oncol..

[126] Zhou X., Shi J., Zhang X., Ge W., Xu Y. MYO1E Correlates with Immune Cells Infiltration and PD-1/PD-L1 Expression in Ovarian Cancer, 31 July 2023, PREPRINT (Version 1). https://www.researchsquare.com/article/rs-3202355/v1.

[127] Chu H.Y., Chen Z., Wang L., Zhang Z.K., Tan X., Liu S., Zhang B.T., Lu A., Yu Y., Zhang G. (2021). Dickkopf-1: A Promising Target for Cancer Immunotherapy. Front. Immunol..

[128] Kim S.R., Won H.S., Yang J.H., Sun S., Yim K., Hong M., Hong S.A., Yoon J.S., Chun S.H., Kim K. (2022). Prognostic value of Dickkopf-1 and ß-catenin expression according to the antitumor immunity of CD8-positive tumor-infiltrating lymphocytes in biliary tract cancer. Sci. Rep..

[129] Kim T., Johnston J., Castillo-Lluva S., Cimas F.J., Hamby S., Gonzalez-Moreno S., Villarejo-Campos P., Goodall A.H., Velasco G., Cardiogenics Consortium (2022). TRIB1 regulates tumor growth via controlling tumorassociated macrophage phenotypes and is associated with breast cancer survival and treatment response. Theranostics.

[130] Zhang X., Zhang B., Zhang C., Sun G., Sun X. (2021). Current Progress in Delineating the Roles of Pseudokinase TRIB1 in Controlling Human Diseases. J. Cancer.

[131] Xie J., Luo X., Deng X., Tang Y., Tian W., Cheng H., Zhang J., Zou Y., Guo Z., Xie X. (2023). Advances in artificial intelligence to predict cancer immunotherapy efficacy. Front. Immunol..

[132] Gao Q., Yang L., Lu M., Jin R., Ye H., Ma T. (2023). The artificial intelligence and machine learning in lung cancer immunotherapy. J. Hematol. Oncol..

