# Supplementary material for: Prognostic Indicators for Precision Treatment of Non-Small Cell Lung Carcinoma
Source: Cells. 2024 Oct 28;13(21):1785. doi: 10.3390/cells13211785 (PMC11545304; doi:10.3390/cells13211785)
Supplement: Supplementary file 1 [file cells-13-01785-s001.zip › cells-3243475-supplementary.pdf]

SUPPLEMENTARY FIGURES

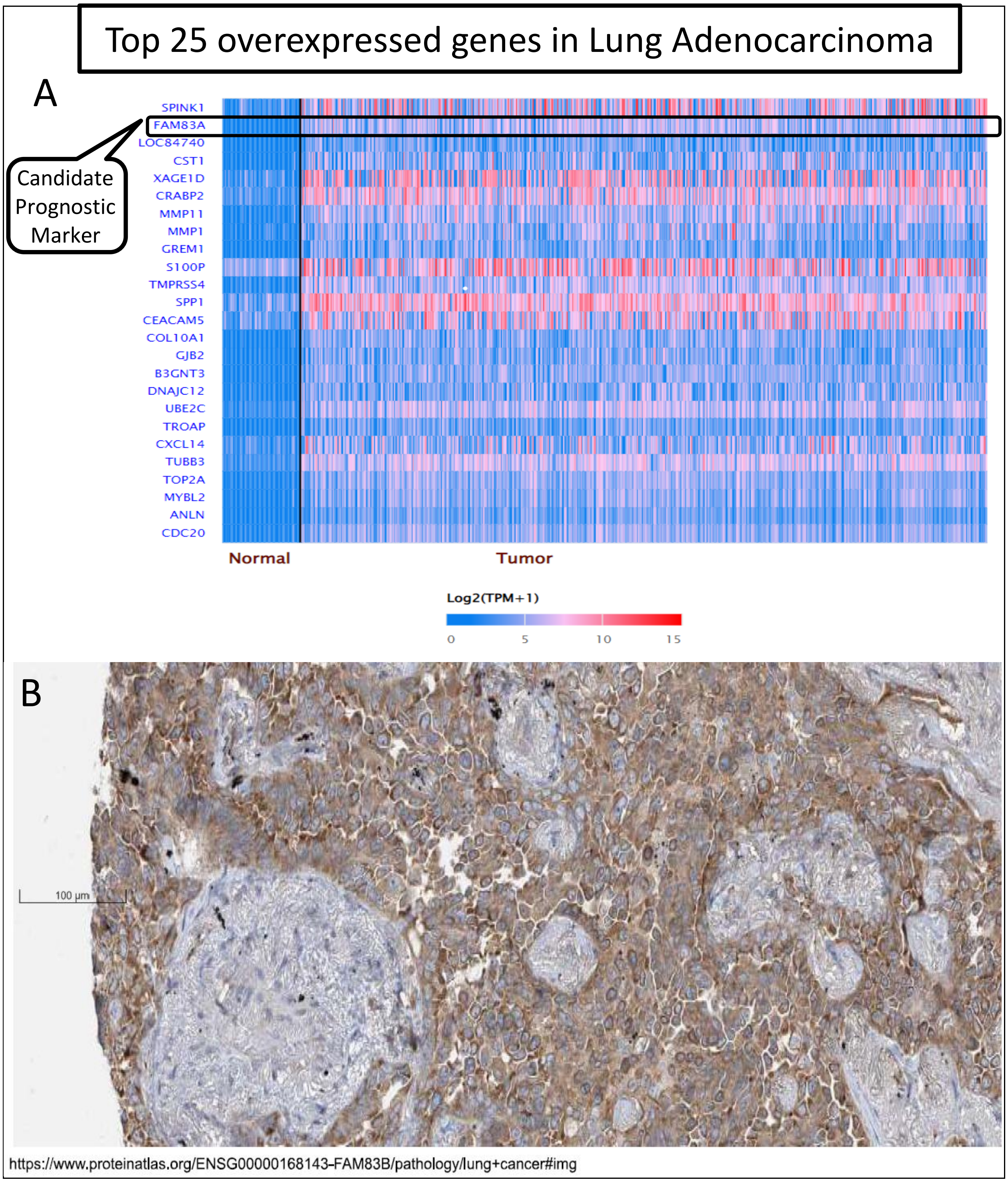

Supplementary Figure S1: Over-expression of FAM83 homologs in NSCLC. (A) Gene expression data from UALCAN. (B) Protein expression data from The Human Protein Atlas. [NSCLC: non-small cell lung carcinoma; TCGA: The Cancer Genome Atlas; UALCAN: University of ALabama at Birmingham CANcer data analysis Portal].

SUPPLEMENTARY FIGURES

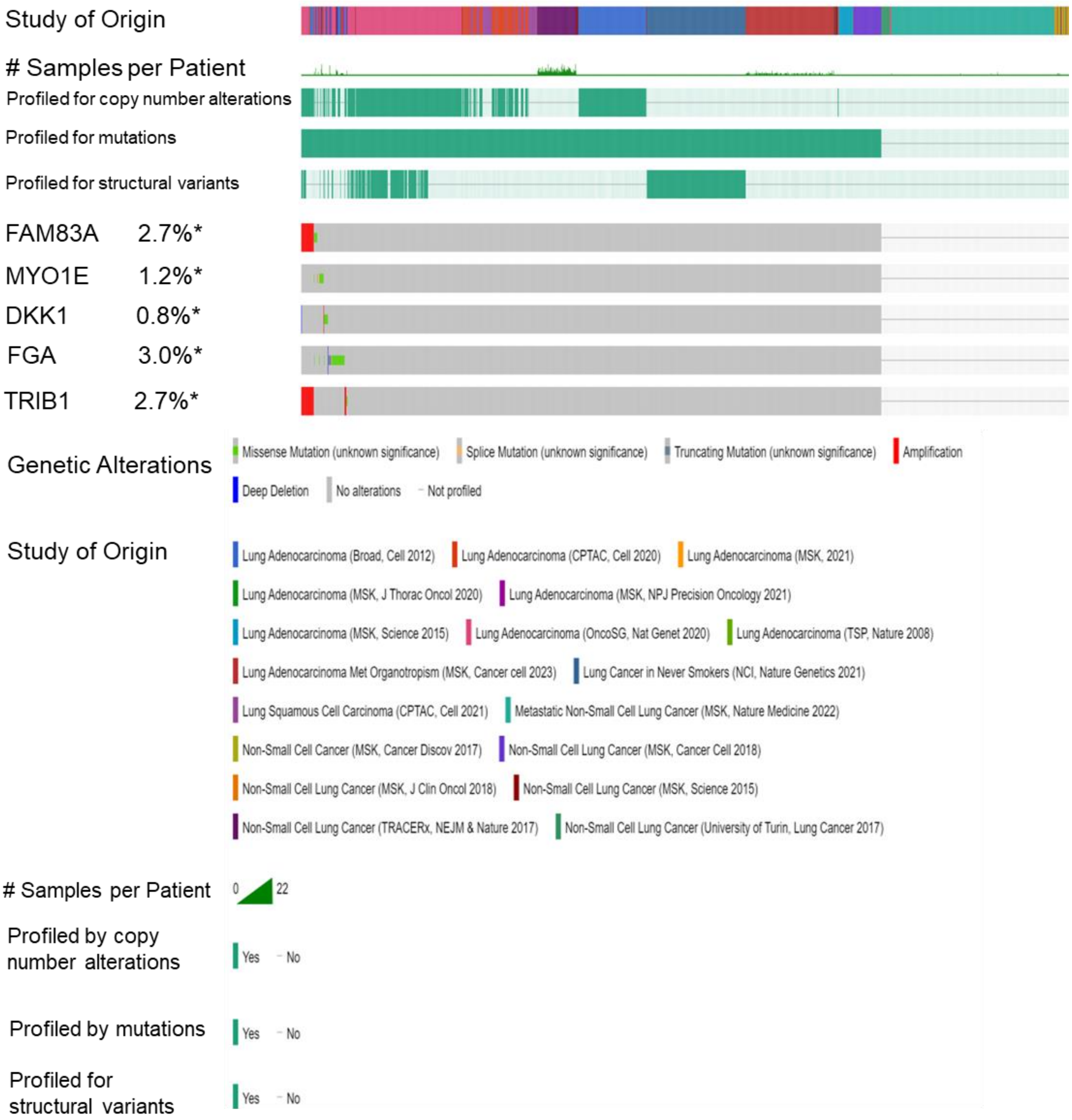

Supplementary Figure S2: Oncoprint: cBioPortal based view of Genomic alterations in NSCLC from non-TCGA cohorts showing amplifications in FAM83A and TRIB1. This is a truncated view of the oncoprint, mainly showing cases harbouring genetic alterations in the studied genes. [Gene expression data were not available].
